# Extracellular Metabolites and Natural Killer Cell Antitumor Function: Mechanisms and Therapeutic Strategies

**DOI:** 10.34133/research.1316

**Published:** 2026-06-09

**Authors:** Ruichen Zang, Chen Zhang, Yutian Fu, Juntong Chen, Yicheng Chen, Yanlan Yu, Bufu Tang, Jie Zhang, Guoqing Ding

**Affiliations:** ^1^ Department of Urology, Sir Run Run Shaw Hospital, School of Medicine, Zhejiang University, Hangzhou, China.; ^2^ Department of Interventional Radiology, Zhongshan Hospital, Fudan University, Shanghai, China.; ^3^ National Engineering Research Center of Innovation and Application of Minimally Invasive Instruments, Sir Run Run Shaw Hospital, School of Medicine, Zhejiang University, Hangzhou, China.

## Abstract

Natural killer (NK) cells are innate cytotoxic lymphocytes capable of eliminating malignant cells independent of prior antigen sensitization, thereby constituting a critical first-line defense in tumor immunosurveillance. Within the tumor microenvironment (TME), metabolic dysregulation profoundly impairs NK cell effector function, and accumulating studies have investigated the regulatory effects of extracellular metabolites on NK cells. This review systematically delineates the direct mechanistic interplay between extracellular metabolites and NK cell antitumor immunity, with particular emphasis on their roles as structural components, bioenergetic substrates, metabolic reprogramming inducers, modification donors, gene transcription regulators, stress response mediators, intracellular pH modulators, and ligands engaging cognate receptors to initiate downstream signaling cascades. Furthermore, we discuss the therapeutic strategies targeting metabolites to potentiate NK cell functionality, encompassing modulation of metabolite availability within the TME and exploitation of metabolite-sensitive signaling axes. In addition, combination of metabolic interventions with other modalities, such as adoptive NK cell transfer and anti-programmed cell death protein 1/programmed death-ligand 1 therapy, is also evaluated for prospective applications. This review provides a conceptual framework for understanding the metabolic regulation of NK cells, highlighting emerging directions for advancing NK-cell-centered cancer immunotherapy through metabolic modulation.

## Introduction

Natural killer (NK) cells are cytotoxic lymphocytes derived from bone marrow (BM) hematopoietic stem cells (HSCs) that can rapidly eliminate infected, allogeneic, or stressed cells without prior antigen exposure. As a critical component of the innate immune system, NK cells participate in various physiological and pathological processes, including promoting angiogenesis at the fetal-maternal interface during pregnancy and sustaining autoimmune responses [1]. Furthermore, NK cells also function as potent antitumor effectors capable of recognizing and eliminating tumor cells of diverse histological origins, thereby contributing to cancer immunosurveillance [2].

Metabolites, including carbohydrates, amino acids, lipids, and their derivatives, are essential biomolecules that serve as fundamental building blocks, energy sources, and function regulators required for biological activities. Metabolites within the tissue microenvironment can be acquired from exogenous sources such as dietary intake and subsequently distributed through the circulatory system or alternatively derived from cellular secretion within the local milieu. For NK cells, extracellular metabolites refer specifically to those present in the microenvironment, as distinct from metabolites generated intracellularly through NK-cell-intrinsic metabolic processes. Within the tumor microenvironment (TME), such metabolites profoundly regulate NK cell antitumor function through diverse direct mechanisms.

At present, multiple strategies have been developed to harness NK cells for cancer therapy. Although these approaches have shown considerable efficacy in both preclinical and clinical studies, their therapeutic efficacy and durability are yet to be fully optimized, underscoring the need for complementary strategies. Considering the regulatory effects of extracellular metabolites on NK cells, modulating NK cell function through metabolite-based interventions represents a promising strategy for NK-cell-centered cancer therapy.

Here, we outline the physiological and functional characteristics of NK cells, with a particular focus on the mechanisms by which extracellular metabolites regulate NK cell antitumor immune response, as well as strategies targeting metabolites and metabolite-sensitive axes to enhance NK cell antitumor function. This review also provides a conceptual framework for the future development of NK-cell-centered metabolite-based antitumor interventions, along with their potential integration with other therapeutic modalities.

## Physiological and Functional Characteristics of NK Cells

NK cells constitute a distinct subset of cytotoxic lymphocytes and play a pivotal role in innate immunity. As a specialized immune population, NK cells undergo a tightly regulated development process to acquire effector functions and sustain effective immune surveillance.

### Development and maturation

Originating from HSCs, NK cells undergo a series of tightly regulated developmental processes to achieve lineage commitment, differentiation, and functional maturation.

The prevailing linear model postulates that NK cells exist as a continuum as follows [3]: Human HSCs differentiate into multipotent progenitors, which subsequently give rise to common lymphoid progenitors that can further develop into NK progenitors and other innate lymphoid cell precursors. NK progenitors subsequently differentiate into immature NK cells, and appearance of cluster of differentiation 56 (CD56) denotes the final transition from immature NK to mature NK cells. Based on CD56 expression levels, mature NK cells are categorized into CD56^bright^CD16^−^ NK cells and CD56^dim^CD16^+^ NK cells. CD56^bright^CD16^−^ NK cells exhibit low cytotoxicity and robust cytokine production upon stimulation, displaying a relatively immature functional profile. By contrast, CD56^dim^CD16^+^ NK cells represent a more mature stage, characterized by potent cytotoxicity. Moreover, recent studies reveal that NK cell development is considerably more intricate than previously appreciated, highlighting the diverse origins of NK cells [4].

### Effector function

Once recruited to target sites, NK cells exert their effector functions principally through 3 mechanisms [2]. First, NK cells form immune synapses with target cells, where cytotoxic granules are polarized and secreted. These cytotoxic granules contain perforin, cathepsin C, and granzyme, which collectively induce lysis and apoptosis of target cells. Second, NK cells secrete a variety of cytokines that induce target cell death and modulate the activity of other immune cells, including interferon (IFN). Third, Fas ligand and tumor necrosis factor (TNF)-related apoptosis-inducing ligand (TRAIL) on NK cells engage their corresponding receptors Fas and TRAILR on target cells, leading to caspase-8 activation and apoptosis of target cells.

Unlike CD8^+^ T cells, activation of NK cells is independent of prior antigen presentation. Instead, NK cell responses are determined by a subtle balance between activating and inhibitory signals transmitted through surface immune receptors, which exhibit cross-talk or synergistic interactions. Properties of these receptors have been comprehensively discussed in previous studies [1,5], and we briefly depict the most canonical ones in Table 1.

**Table 1.** Immune receptors regulating NK cell effector function

	Receptor	Ligand(s)
**Activating**	CD16	Fc portion of immunoglobulin G antibody
	NCR family (NKp46, NKp30, and NKp44)	calreticulin P domain, MLL5, PCNA, and B7-H6
	NKG2D	MIC-A/B and ULBP1-6
	SLAM family (2B4, NTB-A, and CRACC)	CD48 and self-ligands
	DNAM-1	PVR and Nectin-2
**Inhibitory**	KIR	MHC-I
	CD94/NKG2A	Nonclassical MHC-I
	TIGIT	PVR, PVRL2, PVRL3, and PVRL4
	TIM-3	Galectin-9, CEACAM-1, phosphatidylserine, and HMGB1

NK, natural killer; CD16, cluster of differentiation 16; TIM-3, T cell immunoglobulin and mucin-domain containing-3; NCR, natural cytotoxicity receptor; NKp46, natural killer cell p46-related protein; NKG2D, natural killer group 2 member D; SLAM, signaling lymphocytic activation molecule; DNAM-1, DNAX accessory molecule 1; KIR, killer cell immunoglobulin-like receptor; TIGIT, T cell immunoreceptor with immunoglobulin and immunoreceptor tyrosine-based inhibitory motif domains; MLL5, mixed-lineage leukemia protein 5; PCNA, proliferating cell nuclear antigen; B7-H6, B7 homolog 6; ULBP1-6, UL16-binding protein 1-6; PVR, poliovirus receptor; MHC-I, major histocompatibility complex-I; PVRL2, poliovirus-receptor-related protein 2; CEACAM-1, carcinoembryonic antigen-related cell adhesion molecule 1; HMGB1, high mobility group box 1.

## Impact of Extracellular Metabolite on NK-Cell-Mediated Antitumor Immunity

Extracellular metabolites refer to metabolic factors present within the tissue microenvironment that are acquired through external supply and delivered to tissues via systemic circulation or generated locally through metabolic events and secretion of neighboring cells, rather than being synthesized by NK cells themselves. For NK cells, they serve not only as essential building substrates for growth and maintenance, but also as critical energy fuels and signal modulators that shape NK-cell-mediated antitumor immune response. In the followings, we summarize how major classes of extracellular metabolites directly regulate NK-cell-mediated antitumor immune response through diverse mechanisms.

### Carbohydrates and their metabolites

**Fig. 1.**
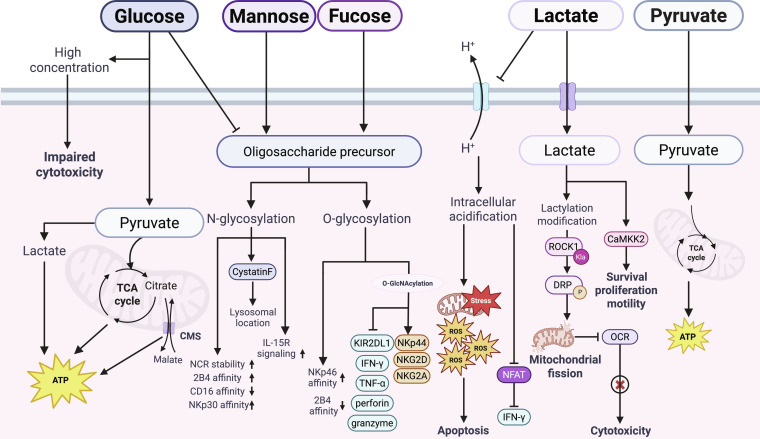
Impact of carbohydrates and their metabolites on natural killer (NK)-cell-mediated antitumor immunity. Extracellular glucose modulates NK cell antitumor functionality by serving as a primary energetic substrate. Other carbohydrates, such as mannose, regulate NK cell activity by providing precursors for glycosylation, thereby influencing protein stability, protein cellular localization, and receptor–ligand affinity. Lactate, the most abundant carbohydrate metabolite within the tumor microenvironment (TME), impairs the antitumor capacity of NK cells by inducing intracellular acidification. Furthermore, upon cellular uptake, lactate can influence NK cells through the lactylation and promotion of their proliferation. Additional carbohydrate-derived metabolites, including pyruvate, also regulate NK cell function via distinct mechanisms.

Carbohydrates represent one of the most abundant metabolites in the microenvironment, playing indispensable roles in sustaining systemic and cellular functions, and can be further converted into diverse metabolites through metabolic processing. For NK cells, carbohydrates and their derived metabolites modulate their antitumor immune response primarily by serving as metabolic fuel, supplying precursors for protein modification, as well as inducing intracellular acidification (Fig. 1).

#### Carbohydrates

##### Glucose as metabolic fuel

Activated NK cells increase glucose uptake, glycolytic rate, and oxidative phosphorylation (OXPHOS) to maximize their effector function. More specifically, excessive glucose consumption by tumor cells depletes glucose in the TME, leading to reduced IFN-γ production and impaired antitumor responses of NK cells [6].

In terms of mechanism, glucose serves as the primary energetic substrate sustaining NK cell metabolism. Upon uptake, extracellular glucose is catabolized through glycolysis into pyruvate, which can either be reduced to lactate via anaerobic glycolysis or enter the mitochondria for further metabolism. Within mitochondria, a portion of pyruvate enters the tricarboxylic acid (TCA) cycle, while the remainder is converted to citrate and participates in the citrate-malate shuttle (CMS), providing an alternative mechanism for mitochondrial nicotinamide adenine dinucleotide (NADH) generation and enhancing glycolytic flux. Unlike TCA cycle, which can utilize multiple fuel sources, CMS relies exclusively on glucose, underscoring the glucose dependence of NK cells [7].

However, in vitro exposure to high concentrations of glucose markedly diminishes NK cell cytotoxicity [8], indicating that extracellular glucose can modulate NK cell function through mechanisms beyond bioenergetic support.

##### Carbohydrates as substrates for protein glycosylation

Upon uptake, carbohydrates such as glucose, mannose, and fucose can be converted into glycosyl donors for N-linked or O-linked glycosylation, thereby modifying multiple function-associated molecules. These glycosylation modifications exert dual effects on NK cell antitumor activity, either enhancing or attenuating immune function depending on the specific targets and context.

N-linked glycosylation directly modulates immune receptor stability and affinity to ligands. Natural cytotoxicity receptors (NCRs) are predicted to possess multiple N-glycosylation sites, and mutations of these sites accelerates degradation of NCRs, indicating that N-glycosylation sustains their stability and thus promotes NK cell activation [9]. Moreover, N-glycosylation of 2B4 is indispensable for its ligand binding, and loss of this modification impairs NK cell cytotoxicity [10]. Likewise, N-glycosylation has also been reported to modulate ligand-binding capacity of natural killer cell p30-related protein (NKp30) and CD16, thereby regulating the antitumor functionality of NK cells [11,12].

N-glycosylation can also modulate NK cell antitumor functionality by modulating the glycosylation status of critical signaling and effector proteins. N-glycosylation is indispensable for the proper lysosomal localization of cystatin F, where it inhibits cathepsin C activity and thereby impairing NK cell cytotoxicity [13]. Furthermore, fucosyltransferase-8 deficiency and consequent core fucosylation impairment down-regulates the expression of proteins associated with interleukin-15 receptor (IL-15R) signaling, NK cell activation, and type I IFN responses, thereby impairing degranulation capacity and IFN-γ production of NK cells, compromising their antitumor immune response [14].

Beyond N-linked glycosylation, O-linked glycosylation also plays a critical role in NK cell receptor regulation. O-glycosylation at Thr225 of NKp46 is essential for tumor recognition [9], while O-glycosylation of 2B4 weakens its binding to CD48 and impairs 2B4-mediated lysis of CD48^+^ tumor targets [10]. O-linked-β-*N*-acetylglucosaminylation, a subtype of O-glycosylation, further modulates NK cell receptor expression and effector molecule production. Pharmacologic inhibition of O-linked-β-*N*-acetylglucosamine (O-GlcNAc) transferase with OSMI-1 down-regulates the expression of activating receptors NKp44 and natural killer group 2 member D (NKG2D), along with inhibitory receptor NKG2A, while up-regulating killer cell immunoglobulin-like receptor 2DL1 (KIR2DL1) and suppressing TNF-α, IFN-γ, perforin, and granzyme secretion, ultimately diminishing NK cell cytotoxicity [15].

#### Carbohydrate-derived metabolites

Beyond modulating NK cells by themselves, carbohydrates can be catabolized into a spectrum of downstream metabolites to modulate diverse aspects of NK cell biology. Among them, lactate is one of the most abundant and extensively studied molecule, while others also contribute to the modulation of NK cell antitumor immune responses.

##### Lactate

Lactate overproduction represents a hallmark metabolic feature of tumor cells, driven by the shift toward glycolysis mediated through the induction of key metabolic enzymes such as lactate dehydrogenase A (LDHA). The excess lactate produced in tumor cells is further exported by monocarboxylate transporters (MCTs), resulting in substantial accumulation of lactate in the TME [16].

Extracellular lactate markedly impairs NK cell viability and functionality. Lactate exposure has been reported to induce NK cell apoptosis, reduce their infiltration within tumors, down-regulate activating receptors such as NKp46, and diminish perforin and granzyme release, thereby impairing NK cell antitumor function [17,18].

Mechanistically, lactate compromises the antitumor functionality of NK cells by inducing their intracellular acidification. Lactate accumulation in the TME results in impaired proton efflux and subsequent intracellular acidification of NK cells, which induces mitochondrial stress and reactive oxygen species (ROS) production, thereby triggering apoptosis [19]. Intracellular acidification also suppresses nuclear factor of activated T cell (NFAT) transcription, thereby attenuating IFN-γ and cytokine production, ultimately impairing NK-cell-mediated antitumor immune responses [17].

Beyond inducing intracellular acidification, lactate can also modulate NK cell antitumor immune response through its uptake by NK cells and subsequent conversion that promotes protein lactylation (Kla). Elevated lactate levels have been reported to increase global lactylation [20], which negatively correlates with tumor infiltration of NK cells [21]. Further study demonstrates that lactate induces Kla of Rho-associated coiled-coil containing protein kinase 1 (ROCK1) at Lys13, promoting phosphorylation of dynamin-related protein 1 (DRP1) at Ser616, resulting in mitochondrial fission and reduced oxygen consumption rate (OCR), thereby dampening NK cell cytotoxicity [22].

However, lactate seems to exert multifaceted effects on NK cells. Lactate uptake via MCT1 by NK cells induces calcium/calmodulin-dependent protein kinase kinase 2 (CaMKK2) expression in NK cells and enhances NK cell survival, proliferation, and motility without directly affecting their cytotoxicity [23], potentially representing an adaptive mechanism that enables NK cells to function within lactate-enriched TME.

##### Other carbohydrate-derived metabolites

Apart from lactate, other carbohydrate-derived metabolites also contribute to the regulation of NK-cell-mediated antitumor immune responses.

Pyruvate is the terminal product of glycolysis, and exogenous pyruvate has been demonstrated to maintain NK cell metabolism through fueling TCA cycle. Endogenous glycolysis-derived pyruvate is limited and preferentially directed toward serine biosynthesis, resulting in insufficiency of TCA cycle substrate. Thus, uptake of extracellular pyruvate serves as an indispensable energy resource to maintain bioenergetic metabolism, adenosine triphosphate (ATP) content and NAD level of NK cells, thereby supporting their viability and functionality [24].

### Amino acids and their metabolites

**Fig. 2.**
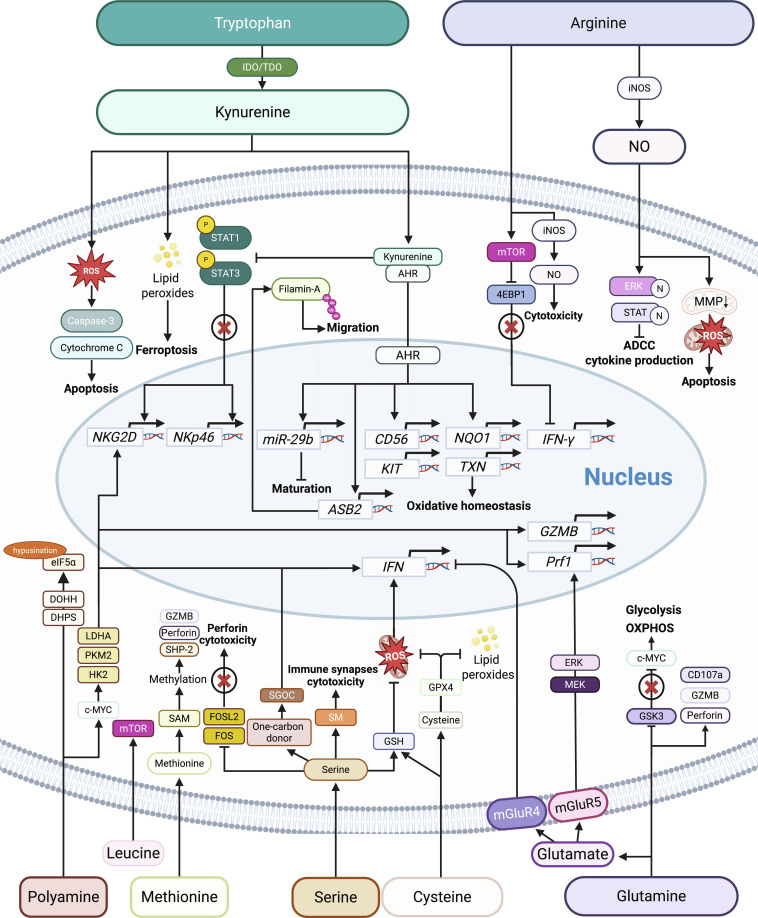
Impact of amino acids and their metabolites on natural killer (NK)-cell-mediated antitumor immunity. Distinct amino acids modulate the antitumor function of NK cells through diverse molecular mechanisms. Briefly, amino acids and their metabolites can modulate NK cell antitumor immunity by binding to specific receptors of NK cells such as aryl hydrocarbon receptor (AHR) and metabotropic glutamate receptors (mGluRs) and activating subsequent signaling pathways, maintaining activation-associated metabolic reprogramming, preserving intracellular redox homeostasis, and supplying precursors for the biosynthesis of other cellular components. Ub, ubiquitin.

Amino acids and their downstream metabolites play multifaceted roles in vital physiological processes, including energy metabolism and signal transduction. For NK cells, amino acids and their metabolites modulate antitumor immunity primarily by supplying precursors for the biosynthesis of other cellular components, inducing metabolic reprogramming, supplying precursors for RNA modification, maintaining intracellular redox homeostasis, and binding to specific receptors on NK cells and subsequently initiating associated signaling pathways (Fig. 2).

#### Kynurenine

Kynurenine is derived from tryptophan by indoleamine 2,3-dioxygenase (IDO) and tryptophan 2,3-dioxygenase (TDO) [25] and regulates NK cell antitumor immune responses through multiple mechanisms, predominantly exerting immunosuppressive effects.

Kynurenine suppresses NK-cell-mediated antitumor immunity by impairing their survival and proliferation. Exposure to kynurenine elevates ROS level, activates caspase-3, and triggers cytochrome c release in NK cells, ultimately culminating in apoptosis [26]. Kynurenine also promotes the accumulation of lipid peroxides in NK cells, leading to ferroptosis and impaired tumor infiltration of NK cells [27].

Kynurenine is also a ligand of aryl hydrocarbon receptor (AHR) and can modulate NK cell antitumor immunity through AHR activation. AHR regulates NK cell development and maturation by binding to the enhancer region of *miR-29b*, as well as the promoters of *CD56* and *KIT*, thereby enhancing their transcription [28,29]. AHR activation also enhances NK cell migration by binding to the promoter of *ASB2*, thereby increasing ASB2 expression and promoting ASB2-dependent filamin-A proteasomal degradation [30]. Moreover, by activating AHR, kynurenine can also regulate NK cell functionality. Kynurenine-induced AHR activation reduces the phosphorylation of signal transducers and activators of transcription 3 (STAT3) and STAT1, leading to decreased expression of NKG2D and NKp46 and consequent impairment of NK cell cytotoxicity [31]. Kynurenine-induced AHR activation also up-regulates nicotinamide adenine dinucleotide (phosphate) hydrogen [NAD(P)H], NAD(P)H quinone dehydrogenase-1 (NQO1), and thioredoxin (TXN) expression, thereby modulating oxidative stress and cellular metabolism, ultimately inhibiting NK cell cytotoxicity [28].

#### Arginine-NO axis

Arginine depletion inhibits NK cell antitumor functionality through multiple ways. Arginine is the principle metabolic activator of mammalian target of rapamycin (mTOR) pathway, and reduced arginine availability weakens mTOR activation in NK cells, which, in turn, increases eukaryotic translation initiation factor 4E-binding protein 1 (4EBP1) activity and restrains translation of IFN-γ transcripts, subsequently diminishing IFN-γ production and impairing NK cell antitumor immune response [32].

Moreover, arginine can also be converted into nitric oxide (NO) by inducible NO synthase (iNOS) in NK cells, and NK-cell-derived NO synthesis has been proposed to act as an accessory cytotoxic mechanism that contributes to DNA fragmentation and lysis of target cells. While arginine supplementation markedly enhances the proportion of tumor-infiltrating and cytotoxic NK cells, as well as suppresses tumor growth in *Inos*^fl^/^fl^Ctrl mice, NK-cell-specific *Inos* deletion completely abrogates these effects, demonstrating that arginine in the TME critically potentiates NK-cell-mediated antitumor immunity by augmenting intra-NK-cell NO synthesis [33].

However, arginine can also be converted to NO by iNOS extracellularly, which negatively modulates NK-cell-mediated antitumor immune responses. Mesenchymal stem cell (MSC) conditioned medium impairs NK cell cytotoxicity, and the suppressive effects are abrogated by iNOS inhibition, suggesting that MSC-derived iNOS and associated NO accumulation contributes to the impairment of NK cell antitumor responses [34]. In terms of mechanism, the inhibitory effects of extracellular NO exert on NK cells are closely associated with protein nitration. Exposure to NO induces nitration of signaling proteins in NK cells, such as extracellular signal-regulated kinase (ERK) and STAT, thus inhibiting Fc-receptor-activation-induced activating signaling and leading to impairment in NK cell effector functions, including antibody-dependent cellular cytotoxicity (ADCC) and cytokine production [35]. Moreover, extracellular NO also decreases mitochondrial membrane potential (MMP) of NK cells, thereby promoting the elevation of ROS levels and NK cell apoptosis [36].

#### Glutamine-glutamate axis

Glutamine availability can enhance NK-cell-mediated antitumor immunity through support of proliferation and functional competence. Restricted glutamine availability to NK cells resulting from elevated glutamine uptake by colorectal cancer (CRC) cells markedly decreases expression of CD107a, perforin, and granzyme B (GZMB) in NK cells, increases apoptosis of intratumoral NK cells, and ultimately impairs NK cell antitumor immunity [37]. Mechanistically, glutamine promotes NK cell antitumor function mainly by maintaining their metabolic reprogramming. Upon uptake by NK cells, glutamine stabilizes c-Myc expression, thereby sustaining the enhancement of glycolysis and OXPHOS following NK cell activation. Consistently, glutamine deprivation increases glycogen synthase kinase 3 (GSK3)-mediated c-Myc degradation, leading to impaired NK cell metabolic reprogramming, decreased IFN-γ and GZMB expression, and substantially compromised NK-cell-mediated antitumor immune responses [38].

Glutamine can be metabolized to glutamate, which can also be taken up from the extracellular. Acting as a ligand for metabotropic glutamate receptors (mGluRs), glutamate activates these receptors and modulates NK cell antitumor functionality. NK cells primarily express mGluR1, mGluR3, mGluR4, mGluR5, and mGluR8, among which mGluR1 and mGluR5 are activating receptors, whereas mGluR3, mGluR4, and mGluR8 are inhibitory receptors [39,40]. Activation of mGluR5 by glutamate increases expression of GZMB and perforin in NK cells via the mitogen-activated protein kinase (MAPK) kinase/ERK signaling pathway, thereby augmenting NK cell cytotoxicity and tumor infiltration [39,40]. Conversely, mGluR4-deficient NK cells displays markedly elevated Ki-67 expression, enhanced transcriptional activation, and increased IFN-γ production, implying the inhibitory role of glutamate-induced mGluR4 activation in NK cell antitumor immunity [41].

#### Cysteine-glutathione axis

Cysteine is transported into NK cells through neutral amino acid transporters and subsequently utilized by γ-glutamylcysteine synthetase for glutathione (GSH) synthesis [42].

Acting as a direct substrate for GSH peroxidase 4 (GPX4), cysteine counteracts lipid peroxidation and stress-induced ferroptosis in NK cells [42]. Likewise, decrease in intracellular GSH levels in NK cells disrupts redox homeostasis and elevates mitochondrial ROS level, resulting in mitochondrial dysfunction and impaired OXPHOS, thereby diminishing NK cell proliferation and cytotoxic capacity [43].

#### Serine

Serine contributes to NK cell antitumor functionality by providing precursor for crucial biomolecules. Serine deficiency impairs NK cell sphingolipid synthesis and cellular sphingomyelin (SM) level, as well as membrane protrusions formation, thereby diminishing NK cell cytolytic immune synapses and cytotoxicity [44]. Serine also participates in the serine-glycine-one carbon (SGOC) pathway as a one-carbon donor, promoting the generation of downstream one-carbon metabolites that sustain NK cell proliferation and IFN production. Moreover, serine also contributes to the synthesis of GSH and maintenance of mitochondrial ROS homeostasis, thereby enhancing IFN production in NK cells [45].

However, it is also reported that extracellular serine down-regulates c-Fos (FOS) and Fos-related antigen 2 (FOSL2) in NK cells, which are 2 key transcription factors involved in the MAPK signaling pathway, thereby suppressing the expression of functional molecules and cytotoxicity of NK cells [46]. Thus, the dual role of serine underscores the need for a more nuanced investigation into underlying mechanisms.

#### Other amino acids

Beyond the aforementioned amino acids, other amino acids also regulate NK-cell-mediated antitumor immune response.

Methionine can be converted to S-adenosylmethionine (SAM), providing substrates for methylation and regulating NK-cell-mediated antitumor immune responses [47]. For instance, the gene encoding Src homology region 2 domain-containing phosphatase 2 (SHP-2) in NK cells is m^6^A modified, and deficiency of m^6^A writer METTL3 notably decreases SHP-2 expression, thereby rendering NK cells hyporesponsive to IL-15, which is associated with suppressed activation of the AKT and MAPK signaling pathways [48]. Moreover, effector function genes of NK cells, such as *Prf1* and *Gzmb*, also requires methylation to sustain NK cell homeostasis, maturation, and antitumor immunity following activation [49], highlighting methionine-derived methylation as a critical mechanism governing NK cell antitumor immunity.

Moreover, leucine can activate the metabolic sensor mechanistic target of rapamycin complex 1 (mTORC1), and leucine deprivation distinctly suppresses NK-cell-mediated antitumor functionality via mTORC1 impairment [38].

#### Polyamines

Polyamines can be acquired exogenously, and 3 major polyamines identified in mammalian cells are putrescine, spermidine, and spermine [50].

Polyamines regulate NK cell functionality to modulate their antitumor immunity. Tumor-induced depletion of polyamines reduces c-Myc and subsequent hwxokinase 2 (HK2), pyruvate kinase M2 (PKM2), and LDHA expression of NK cells and disrupts NK cell metabolic reprogramming, thereby compromising NKG2D, GZMB, and IFN-γ expression and diminishing NK cell cytotoxicity [50]. Moreover, polyamines can also be converted into substrates for hypusination of eukaryotic translation initiation factor 5a (eIF5A) by deoxyhypusine synthase (DHPS) and deoxyhypusine hydroxylase (DOHH), thus regulating eIF5A activity and NK cell function. Notably, treatment of NK cells with DHPS inhibitor GC7 impairs NK cell growth, proliferation, and cytotoxicity [51].

### Lipids and their metabolites

**Fig. 3.**
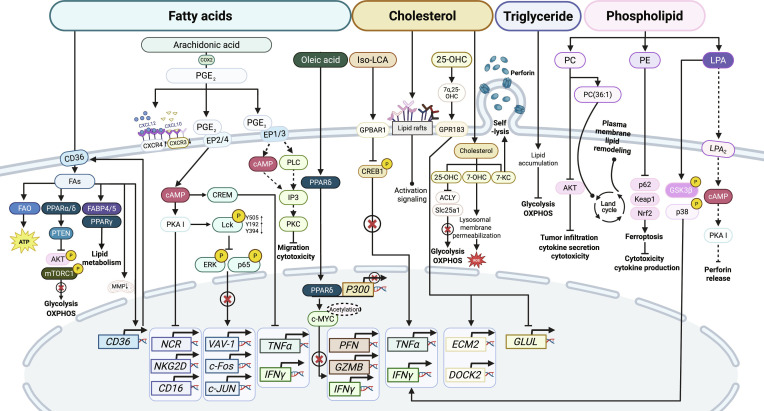
Impact of lipids and their metabolites on natural killer (NK)-cell-mediated antitumor immunity. Fatty acids, cholesterol, triglycerides, and phospholipids modulate NK cell antitumor functionality through multifaceted ways. They can supply metabolic fuel and induce metabolic reprogramming, engage specific receptors to activate downstream signaling pathways, promote the formation of membrane-associated structures, modulate redox homeostasis, and remodel plasma membrane order.

Lipids are a class of biologically diverse molecules characterized by hydrophobic or amphipathic properties. They serve not only as the principal structural components of biological membranes but also as key forms of energy storage and signaling mediators. Lipids are primarily categorized into fatty acyls, glycerolipids, glycerophospholipids, sphingolipids, sterol lipids, prenol lipids, saccharolipids, and polyketides and can be further metabolized into bioactive derivatives such as prostaglandins and oxysterols. Lipids and their metabolites regulate the antitumor immune functions of NK cells by functioning as structural component, supplying metabolic fuel, inducing metabolic reprogramming, engaging specific receptors to activate downstream signaling pathways, as well as modulating redox homeostasis. (Fig. 3).

#### Fatty acids

Fatty acids (FAs) can fuel NK cells by serving as substrates for FA oxidation (FAO). Activated NK cells exhibit increased FAs uptake, and those capable of generating energy from FAs display enhanced cytotoxicity marker up-regulation and target-cell killing capacity [52]. Peroxisome-proliferator-activated receptor γ (PPARγ) and FA-binding protein 4/5 (FABP4/5) are key regulators of lipid metabolism, and inhibition of PPARγ or FABP4/5 suppresses lipid metabolism and IFN-γ expression in activated NK cells, whereas PPARγ agonist rosiglitazone markedly increases IFN-γ production [53].

However, in general, FAs attenuate NK-cell-mediated antitumor immune responses. Treatment with free FAs (FFAs) markedly impairs NK cell proliferation and decreases IFN-γ and GZMB expression in NK cells [54]. Moreover, when FAs are transferred from tumor cells to NK cells, the proportion of CD56^+^IFN-γ^+^GZMB^+^ NK cells decreases, leading to diminished NK-cell-mediated cytotoxicity in coculture system [55].

Mechanism underlying is that FAs can also reprogram NK cell metabolism and suppress NK-cell-activation-related signaling pathways. FFAs can activate PPARα/δ and induce expression of lipid metabolism-associated genes such as PPARδ and FABP4/5, thereby driving NK cells toward lipid metabolism. Meanwhile, FFAs inhibit the phosphorylation of mTORC1 regulatory factor AKT and mTORC1 itself, thereby impairing the ability of NK cells to up-regulate glycolysis and OXPHOS upon stimulation. These metabolic defects result in reduced ATP generation and decreased IFN production and cytotoxicity, and ultimately prevent NK cells from polarizing the microtubule-organizing center (MTOC) and lytic granules toward tumor cells, as well as subsequent degranulation at the immune synapse [53,54]. Further study refines this mechanism, showing that FFA-uptake-driven PPAR activation induces phosphatase and tensin homolog (PTEN) expression, thereby inhibiting the phosphatidylinositol 3-kinase (PI3K)–AKT–mTOR/forkhead box O1 (FOXO1) signaling axis and impairing glycolysis in NK cells. In addition, FA uptake up-regulates CD36 expression, promoting enhanced lipid influx and forming a positive feedback loop that reinforces metabolic reprogramming toward lipid metabolism [56]. Thus, canonical-activation-related metabolic reprogramming such as glycolysis and OXPHOS of NK cells is disrupted, and NK-cell-mediated antitumor immune response is compromised.

FAs can also modulate NK cell antitumor immunity by regulating mitochondrial function. In Eμ-myc lymphoma, which is abundant in FAs, NK cells exhibit markedly reduced mitochondrial mass and membrane potential, indicating mitochondrial impairment [53].

Although collectively referred to as FAs, distinct FAs and their metabolic derivatives exert diverse effects on NK cell regulation. In the following sections, the regulatory mechanisms through which distinct FAs and their metabolites modulate NK cells are discussed.

##### Prostaglandin E_2_

Prostaglandin E_2_ (PGE_2_) is converted by arachidonic acid through cyclooxygenase-2 (COX-2) [57] and acts locally on neighboring cells to exert effects.

PGE_2_ regulates NK cell antitumor functionality at multiple levels. Treatment of PGE_2_ suppresses NK cell proliferation, promotes NK cell apoptosis, and inhibits NK cell maturation [58,59]. PGE_2_ can also alter NK cell migratory mode, and for following PGE_2_ exposure, C-X-C chemokine receptor type 3 (CXCR3) expression on NK cells is down-regulated, whereas CXCR4 expression is up-regulated, thereby reducing NK cell responsiveness to C-X-C motif chemokine ligand 10 (CXCL10) and enhancing NK cell responsiveness to CXCL12 [60]. PGE_2_ also suppresses NK cell functionality. PGE_2_-treated NK cells display reduced surface expression of activating markers, increased surface expression of inhibitory receptors, impaired cytokine production, diminished GZMB and perforin production, as well as attenuated cytotoxicity [57,59]. Moreover, PGE_2_ also delays the polarization of MTOC toward immune synapse, impairs lytic granule release, and lowers both the frequency and kinetics of tumor cell killing [60], resulting in impaired NK-cell-mediated antitumor immunity.

Studies investigating PGE_2_ regulation of NK cells have mainly centered on its activation of E-prostanoid (EP) receptors. EPs are G-protein-coupled receptors comprising 4 subtypes (EP1 to EP4) that share structural similarity but engage distinct intracellular signaling pathways. At present, most investigations have concentrated on the PGE_2_-EP2/EP4 signaling axis. Upon PGE_2_ binding to EP2 or EP4, intracellular adenylyl cyclase is activated, leading to elevated cyclic adenosine monophosphate (cAMP) level and activation of type I protein kinase A (PKA I). PKA I activation subsequently inhibits early signaling proteins in cytotoxic lymphocytes, resulting in down-regulation of NCRs, NKG2D, and CD16, as well as reduced NK cell cytotoxicity [61]. It is further revealed that PKA I-mediated phosphorylation of Lck at Y505 and Y192 alters conformation of Lck, preventing its activation-associated Y394 phosphorylation. Consequently, Lck-dependent NK cell activation cascades, including ERK and p65 phosphorylation, are suppressed, accompanied by down-regulation of VAV guanine nucleotide exchange factor 1 (VAV-1), c-Fos, and c-Jun, thereby leading to impairment in NK cell antitumor functionality [59,62]. Moreover, PGE_2_-induced EP2 /EP4 activation increases cAMP-responsive element modulator (CREM) expression via cAMP signaling pathway and impairs chromatin remodeling of genes associated with chemokine and cytokine signaling, which prevents promoter regions from adopting an open configuration, consequently decreasing chromatin accessibility and transcription, and ultimately compromising NK-cell-mediated antitumor immunity [57]. In contrast, PGE_2_-EP1/3 engagement promotes NK cell migration while suppressing cytokine secretion and cytotoxicity, effects that are likely mediated by alterations in cAMP levels or EP1-driven phospholipase C (PLC) activation, which causes downstream release of inositol 1,4,5-trisphosphate (IP3) and activation of protein kinase C [63].

##### Oleic acid

Oleic acid, a monounsaturated FA, has been reported to play vital biological roles in energy provision and cellular signaling.

Several studies indicate that oleic acid suppresses NK cell antitumor function. It has been demonstrated that oleic acid treatment activates PPARδ in NK cells and promotes the enrichment of PPARδ at the *P300* promoter, thereby suppressing *P300* transcription. Consequently, reduced P300-mediated acetylation of c-Myc leads to its destabilization and decreased abundance, thereby diminishing c-Myc binding to the promoters of *IFN*, *PFN1*, and *GZMB*. This further diminishes P300 recruitment mediated by c-Myc as a sequence-specific transcription factor and decreases subsequent H3K27 acetylation at these promoters, thereby transcriptionally repressing the expression of IFN-γ, perforin, and GZMB, ultimately attenuating the antitumor functionality of NK cells [64].

Conversely, there also exist studies suggesting that oleic acid enhances NK cell antitumor immunity. Deficiency of myocyte enhancer factor 2C (MEF2C) disrupts lipid metabolism and impairs NK cell activity, whereas supplementation with oleic acid restores cytotoxic function in MEF2C-deficient NK cells. Furthermore, administration of oleate alone can increase NK cell cytotoxicity [65]. Therefore, the precise regulatory mechanisms and overall impact of oleic acid on NK-cell-mediated immune responses remain to be further elucidated.

#### Cholesterol and their metabolites

Cholesterol, a type of sterol, is an essential component of membrane-associated structures and serves as the precursor for oxysterols and bile acids.

Cholesterol enhances NK cell antitumor immune responses by promoting the formation of membrane-associated structures. Lipid rafts are lipid structures rich in cholesterol, sphingolipids, and gangliosides, in which cholesterol is presumed to act as a spacer connecting sphingolipids within the phospholipid bilayer of the cell membrane [66]. Lipid rafts on the cytomembrane can recruit NK-cell-activation-related molecules, thereby regulating NK cell function. NK cells isolated from tumor-bearing mice with high serum cholesterol levels exhibit augmented cholesterol accumulation and lipid raft formation, increased immune signal activation, and activating receptors expression, as well as enhanced cytokine and chemokine production. Consequently, these primed NK cells display potent antitumor functionality against both primary hepatocellular carcinoma and its metastases [67]. Similarly, cholesterol uptake by CD16^+^ neutrophils in CRC restricts cholesterol availability for NK cells, thereby reducing lipid raft formation and expression of NCR1 and NKG2D on these rafts, which leads to blocked receptor signaling and subsequent impairment in the production of TNF-α, perforin, granzyme, and IFN-γ, as well as NK cell cytotoxicity [68]. Furthermore, cholesterol also facilitates the establishment of mature immune synapses in NK cells, thereby enabling efficient and targeted killing of tumor cells [69].

Cholesterol can also undergo enzymatic or nonenzymatic oxidation to generate oxysterols, which exert diverse effects on NK cell antitumor functions. Certain oxysterols directly compromise NK cell viability. 7-ketocholesterol (7-KC) reduces cytomembrane-accessible cholesterol in NK cells and, in turn, weakens the protective presynaptic lipid membrane at the NK cell immune synapse, thereby rendering NK cells susceptible to perforin-mediated self-lysis following degranulation [70]. Similarly, 7-hydroxycholesterol (7-OHC) induces NK cell death by triggering early lysosomal membrane permeabilization, followed by oxidative stress [71]. Oxysterols also impair NK cell function through alternative ways. 25-Hydroxycholesterol inhibits sterol regulatory element-binding protein (Srebp) activation, down-regulates the expression of ATP-citrate lyase (ACLY) and solute carrier family 25 member 1 (Slc25a1), and impairs glycolysis and OXPHOS in NK cells, thereby suppressing cytokine-induced IFN-γ production and GZMB expression and diminishing NK cell cytotoxicity [7]. In contrast to these immunosuppressive effects, 7α,25-dihydroxycholesterol (7α,25-OHC), a downstream metabolite of 25-hydroxycholesterol, exerts a stimulatory effect by binding to its receptor G-protein-coupled receptor 183 (GPR183) on NK cells. This interaction up-regulates adhesion-related genes (such as *ECM2*) and membrane organization genes (such as *DOCK2*), while down-regulating metabolic genes (such as *GLUL*) of NK cells, thereby enhancing NK cell tumor infiltration and antitumor capacity [72].

Bile acids, another class of cholesterol-derived metabolites, are synthesized in the liver and further metabolized by the gut microbiota. Iso-lithocholic acid (iso-LCA), one of the secondary bile acids, binds to G-protein-coupled bile acid receptor 1 (GPBAR1) and suppresses the phosphorylation of cAMP-response-element-binding protein 1 in NK cells, a transcription factor that governs cytokine-related gene expression. This inhibition reduces IFN-γ and TNF-α production, attenuates NK cell cytotoxicity, and induces NK cell apoptosis, thereby impairing intratumoral NK-cell-mediated antitumor immune responses [73].

#### Triglycerides

Triglycerides are a major component of glycerides and the principal form of neutral lipids, and proper levels of neutral lipids are essential for NK cell function. NK cells in ovarian cancer ascites, burdened with excessive lipid accumulation, lose their intracellular buffering and storage capacity for neutral lipids, which restricts their antitumor immune response [74]. Similarly, NK cells in the lungs of patients with breast cancer accumulate intracellular lipids after taking up neutral lipids from exosome-like vesicles secreted by metastatic cancer cells, leading to impaired glycolysis and OXPHOS [75]. Consequently, NK-cell-mediated antitumor immune response is compromised by the excessive neutral lipids.

#### Phospholipids

Phospholipids are essential components of biological membranes and play crucial roles in signal transduction, with different phospholipid subtypes exerting distinct mechanistic effects on NK cells.

Phosphatidylcholine (PC) is the most abundant phospholipids, and is the main component of lecithin. PC has been reported to inhibit the AKT pathway, consequently impairing NK cell tumor infiltration, IFN-γ and GZMB secretion, and cytotoxicity [76]. Moreover, treatment with PC(36,1), a subtype of PC, disturbs NK cell plasma membrane order by altering the transcription of genes involved in plasma membrane lipid remodeling, thereby disrupting the capacity of NK cells to polarize toward target cells, compromising NK-cell-mediated antitumor immune responses [74].

Phosphatidylethanolamine (PE), an effector metabolite produced by ketogenic diet, participates actively in protein biogenesis and functional regulation. By inhibiting the p62–Kelch-like ECH-associated protein 1 (Keap1)–nuclear factor erythroid 2-related factor 2 (Nrf2) pathway and attenuating cellular antioxidant defenses, PE induces ferroptosis in NK cells, leading to cytotoxicity and cytokine production impairment, thereby facilitating the metastatic growth of CRC in the liver [77].

Lysophosphatidic acid (LPA) is another essential extracellular mediator with diverse biological functions. While LPA activates GSK3β and p38 in NK cells and enhances IFN-γ production, it can also suppress perforin release from NK cells against melanoma cells in a dose-dependent manner. This inhibitory effect is likely mediated by the binding of LPA to its receptor LPA2, which triggers the release of the regulatory subunit of PKA I via cAMP pathway. In addition, LPA also regulates NK cell chemotaxis by activating G_i_-dependent transient reorganization of the actin cytoskeleton [78].

### Integrated view

Carbohydrates, amino acids, and lipids represent 3 distinct yet interconnected classes of metabolites, each comprising multiple subcategories. Owing to their unique molecular properties, these extracellular metabolites and their derivatives modulate NK cell antitumor activity directly through diverse mechanisms. Nevertheless, when examined from a holistic perspective, 9 principal regulatory mechanisms can be identified:

• Structural component: Extracellular metabolites can be taken up by NK cells and incorporated as structural components. Cholesterol, for example, serves as a critical constituent of lipid rafts in NK cells and plays an essential role in the formation of immune synapse [68,69].
• Bioenergetic fuel: Extracellular metabolites can be taken up by NK cells and metabolized to fuel cellular bioenergetics. Glucose generates ATP through aerobic glycolysis and TCA cycle, while pyruvate and FAs provide energy via TCA cycle and FAO, respectively [7,24,52].
• Metabolic reprogramming: Extracellular metabolites can be taken up by NK cells and reprogram their metabolic patterns. For instance, glutamine enhances NK cell glycolysis and OXPHOS through c-Myc stabilization, while FAs suppress glycolysis and promote a metabolic shift toward FAO [38,56].
• Protein modification: Extracellular metabolites can be taken up by NK cells and converted into substrates for posttranslational modification. For instance, carbohydrates such as glucose, mannose, and fucose can be converted into glycosyl donors for N-linked or O-linked glycosylation [10]
• RNA modification: Extracellular metabolites can be taken up by NK cells and converted into substrates for RNA modification. Methionine, notably, can be converted to SAM, which serves as a methyl donor for the methylation of genes such as *SHP-2*, *perforin*, and *Gzmb* [47–49].
• Transcriptional regulation: Extracellular metabolites can also be taken up by NK cells and regulate gene transcription. Lactate transported via MCT1 induces CaMKK2 expression in NK cells, whereas PGE_2_ down-regulates CXCR3 expression and up-regulates CXCR4 expression [23,60].
• Cellular stress response: Extracellular metabolites can modulate stress responses in NK cells. Kynurenine exposure, for instance, elevates ROS levels in NK cells, whereas GSH uptake helps maintain intracellular redox balance [26,42].
• pH modulation: Extracellular metabolites can influence environmental pH and thereby regulate intracellular pH in NK cells. Extracellular lactate, for example, inhibits proton efflux from NK cells, leading to their intracellular acidification [19].
• Ligand-receptor interaction: Extracellular metabolites can also act as ligands that bind to receptors of NK cells. Kynurenine binds to and activates AHR, and PGE_2_ serves as a classical ligand for EP receptors [31,62].

**Fig. 4.**
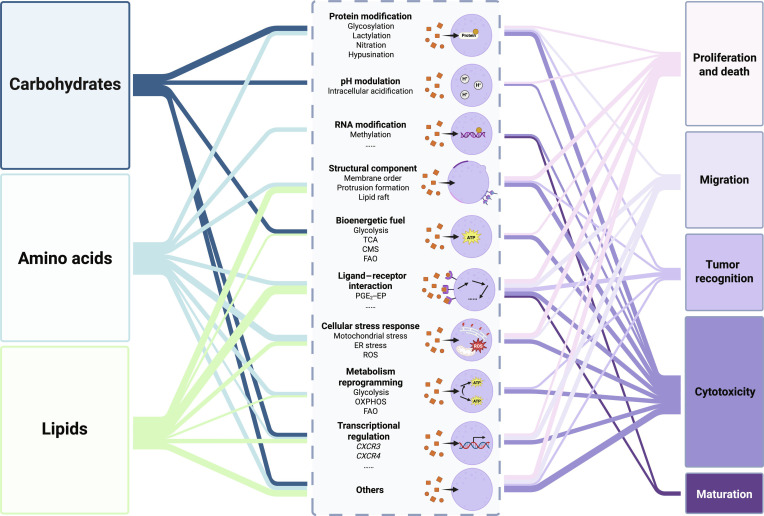
Overarching principles of extracellular metabolite regulation on natural killer (NK) cells. Although different extracellular metabolites regulate NK cell antitumor function through distinct mechanisms, from a holistic perspective, these mechanisms can be categorized into 9 major types: structural component, bioenergetic fuel, metabolic reprogramming, RNA modification, protein modification, cellular stress response, ligand–receptor interaction, and pH modulation, along with others. In addition, several other mechanisms remain to be further elucidated. Through these mechanisms, extracellular metabolites regulate NK cell proliferation and death, maturation, migration, tumor recognition, and cytotoxicity, ultimately shaping NK cell antitumor capacity. ER, endoplasmic reticulum.

Through these 9 principal mechanisms, along with others that are not yet fully characterized, extracellular metabolites shape NK cells in terms of proliferation and death, maturation, migration, tumor recognition, and cytotoxicity, ultimately regulating their antitumor functionality (Fig. 4).

## Tumor Therapeutic Strategies Targeting Extracellular Metabolite for NK Cell Regulation

TME is characterized by nutrient scarcity and waste accumulation, and antitumor immune response of NK cells is tightly regulated by the distinctive metabolite profile [79]. Accordingly, these metabolites and associated signaling pathways can serve as potential targets to enhance NK cell functionality. Here, we discuss the current strategies, recent progress, and future directions of metabolite-based modulation therapy in the context of NK-cell-mediated tumor immunotherapy.

### Modulating extracellular metabolite abundance

**Fig. 5.**
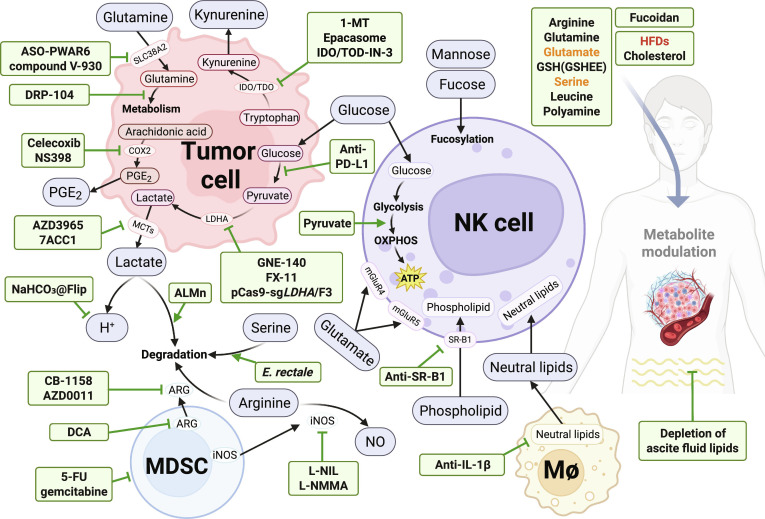
Metabolite-based modulation therapy by regulating extracellular metabolite abundance. Metabolites, including carbohydrates, amino acids, lipids, and their corresponding metabolites, can be acquired from intake and subsequently distributed through the circulatory system to tumor microenvironment (TME) or alternatively derived from cellular secretion within the TME. Interventions that targeting their intake, synthesis, secretion, and degradation can modulate their extracellular abundance, thereby restoring natural killer (NK) cell antitumor functionality. Mø, macrophage.

The concentration of extracellular metabolites can be modulated by altering their uptake amount or biochemical forms, whereas the levels of their downstream metabolites can be regulated through recalibrations in their biosynthesis, secretion, or degradation. This section delineates strategies to manipulate the availability of extracellular metabolites with the aim of augmenting NK-cell-mediated antitumor immune responses (Fig. 5).

#### Carbohydrates and their metabolites

##### Carbohydrates

Enhancing the availability of glucose to NK cells can improve NK-cell-mediated antitumor immune responses. Anti-programmed death-ligand 1 (PD-L1) antibodies have been reported to suppress the translation of glycolytic enzymes in tumor cells, thereby attenuating glycolysis and decreasing glucose uptake of tumor cells, which consequently increases the level of available glucose for NK cells within TME [80].

Supplementation with fucose can also enhance NK-cell-mediated antitumor immune responses by promoting fucosylation in NK cells. Fucoidan extracted from *Durvillaea antarctica*, which consists sulfate groups and polysaccharides such as fucose, has been reported to up-regulate CD69 expression and increase the production of IFN-γ, perforin, and GZMB in NK cells, thereby augmenting NK cell cytotoxic activity and suppressing the growth of metastatic TC-1 lung carcinoma [81], which may be associated with improved fucosylation modifications in NK cells.

##### Carbohydrate metabolites

Reducing lactate accumulation in the TME can be achieved by suppressing lactate synthesis in tumor cells. Lactate in tumor cells is primarily generated by LDHA; thus, inhibition of LDHA represents an effective strategy to improve NK-cell-mediated antitumor immune responses. Studies have demonstrated that the LDHA inhibitor GNE-140 enhances the expression of perforin, GZMB, and TNF-α in NK cells when cocultured with pancreatic cancer cells [82]. Intraperitoneal injection of the LDHA inhibitor FX-11 also markedly elevates the proportion of NKG2D-, NKp46-, and DNAX accessory molecule 1 (DNAM-1)-positive NK cells in peripheral blood, accompanied by a distinct reduction in PanO2 tumor volume and weight [82]. In addition, nanoparticles have been developed for *LDHA* gene editing. The lipoplex pCas9-sg*LDHA*/F3, for example, effectively silences *LDHA* expression in tumor cells and decreases lactate production [83].

Reducing lactate accumulation in the TME can also be achieved by inhibiting lactate secretion from tumor cells. Tumor cells export lactate into the TME primarily through MCTs (particularly MCT1 and MCT4); thus, blockade of MCTs constitutes an effective strategy to enhance NK-cell-mediated antitumor immune responses. Inhibition of MCT1 with AZD3965 lowers lactate level in the TME, resulting in tumor growth suppression [84]. Intraperitoneal administration of the MCT1/4 blocker 7ACC1 also substantially increases the proportion of NKG2D-, NKp46-, and DNAM-1-positive NK cells in peripheral blood, elevates NK cell perforin secretion, strengthens NK cell cytotoxic activity, and leads to a marked reduction in tumor volume [82]. However, MCT1 is also expressed on NK cells and mediates their lactate uptake from the TME. Hence, MCT1 inhibition can also impair NK cell proliferation and migration by attenuating CaMKK2 up-regulation [23]. Therefore, the overall impact of MCT1 inhibition on NK-cell-mediated antitumor immune responses remains to be fully elucidated.

Furthermore, lactate accumulation within TME can be mitigated by enhancing its degradation and consumption. ALMn, a bioresponsive nanoreactor containing lactate oxidase, can degrade lactate through catalytic oxidation and exhibits potent antitumor efficacy [85]. NaHCO_3_@Flip, a novel NaHCO_3_-containing biomaterial, can also neutralize lactate within the TME and restore NK-cell-mediated antitumor immune responses [86].

In addition, supplying pyruvate can energize NK cells. Supraphysiological concentrations of pyruvate can serve as an energy source to sustain NK cell metabolism and enhance NK cell functions in a dose-dependent manner [24].

#### Amino acids and their metabolites

##### Kynurenine

Kynurenine accumulation within the TME can be mitigated by inhibiting the metabolism of tryptophan to kynurenine. IDO1 inhibitor 1-methyl-DL-tryptophan (1-MT) has been reported to restore NK cell cytolytic activity [31]. IDO1 inhibitors can also be structurally optimized to achieve enhanced pharmacokinetic profiles and tumor accumulation, as exemplified by epacasome, which demonstrates superior performance compared with epacadostat [87]. However, inhibition of IDO1 also induces compensatory up-regulation of TDO2 in the TME, thereby compromising the suppressive capacity of IDO1 blockade on kynurenine production. Dual IDO1/TDO2 inhibitor IDO/TDO-IN-3 markedly restores NK cell cytotoxicity suppressed by podoplanin-positive cancer-associated fibroblasts (CAFs) that express both IDO1 and TDO2, highlighting the superiority of dual-target inhibition [25].

##### Arginine-NO axis

The concentration of arginine within TME can be increased by enhancing exogenous arginine uptake. Supplementation with arginine has been shown to enhance NK cell cytotoxicity, cytokine secretion, and tumor infiltration, while reducing the metastatic burden in tumor-bearing mice [33,88].

Arginine availability within TME can also be enhanced by inhibiting arginase (ARG)-mediated catabolism. On one hand, suppression of arginine degradation in the TME can be achieved by reducing the infiltration of ARG-expressing cells and their ARG expression levels. Treatment with dichloroacetate (DCA) decreases ARG expression in tumor-infiltrating immune cells and increases the population of IFN-γ-producing NK cells in the spleens of tumor-bearing mice [89]. On the other hand, inhibition of ARG activity provides another strategy to limit arginine degradation in the TME. CB-1158, a potent and selective inhibitor of extracellular ARG, enhances NK cell infiltration into tumors and up-regulates cytokines and IFN-inducible genes, thereby improving NK-cell-mediated antitumor immune responses [90]. Moreover, AZD0011, a novel peptide boronic acid prodrug acting as an ARG inhibitor, also attenuates tumor growth in an NK-cell-dependent manner [91].

Current NO-related therapeutic approaches primarily aim to reduce extracellular NO synthesis through inhibition of iNOS. On one hand, NO generation in the TME can be suppressed by limiting the infiltration of iNOS-expressing cells. Chemotherapeutic agents such as 5-fluorouracil (5-FU) and gemcitabine can deplete myeloid-derived suppressor cells (MDSCs), which express iNOS, thereby enhancing NK cell cytotoxicity in tumor-bearing mice [35]. On the other hand, the enzymatic activity of iNOS within the TME can be inhibited to decrease NO synthesis. The iNOS inhibitor L-NIL has been demonstrated to enhance the ADCC activity of NK cells against pancreatic cancer cells [35], and L-NMMA has also been reported to restore MSC-conditioned medium-induced impairment of NK cell cytotoxicity [34]. However, iNOS inhibition has been reported to partially impair the antitumor efficacy of NK cells, possibly due to the inadvertent suppression of intracellular NO synthesis within NK cells [92]. Hence, this duality underscores the necessity for a nuanced approach when targeting iNOS to maximize therapeutic benefit.

##### Glutamine–glutamate axis

Enhancing exogenous glutamine uptake can increase glutamine availability within the TME. Long-term oral supplementation of glutamine has been reported to promote NK cell proliferation, enhance NK cell cytotoxicity, and consequently suppress tumor growth [93].

Increase in glutamine availability in TME can also be achieved by inhibiting glutamine uptake in tumor cells. An antisense oligonucleotide-based inhibitor targeting Prader Willi/Angelman region RNA 6 (PWAR6) has been shown to reduce glutamine uptake in tumor cells and enhance glutamine availability for NK cells, thereby promoting NK cell infiltration in both subcutaneous and hepatic tumor burdens [37]. Compound V-930 can selectively block glutamine uptake in cancer cells by inhibiting glutamine transporters [94], which can improve glutamine availability for NK cells. Moreover, although glutamine serves as a critical metabolic substrate supporting tumor cell growth and survival, NK cell metabolism and effector functions remain largely unaffected by glutamine metabolism inhibition. Therefore, targeting glutamine metabolism can selectively block glutamine utilization in tumor cells and increase glutamine availability within the TME without compromising NK cell functionality [38]. For instance, DRP-104, a novel prodrug derivative of the broad-spectrum glutamine antagonist 6-diazo-5-oxo-l-norleucine (DON), inhibits multiple glutamine-utilizing pathways in tumor cells and increases intratumoral glutamine concentrations, thereby promoting NK cell infiltration and proliferation within the TME [95].

Exogenous glutamate supplementation can elevate glutamate levels within the TME, thus modulating the antitumor immune response of NK cells. Subtypes of mGluRs exhibit distinct affinities for glutamate, and the effects of glutamate supplementation on NK-cell-mediated antitumor immune responses vary depending on the administered dose. It is demonstrated that low concentration of monosodium glutamate enhances NK cell functionality, whereas high concentration concurrently activates inhibitory mGluRs and counteracts the stimulatory effects observed at lower doses [40]. Therefore, the optimal glutamate dose for enhancing NK-cell-mediated antitumor immune responses remains to be further investigated.

##### Cysteine-GSH axis

Exogenous GSH supplementation can enhance NK-cell-mediated antitumor immune responses. Oral administration of liposomal GSH, which allows for more efficient absorption of GSH, markedly augments NK cell cytotoxicity [96]. In addition, supplementation with the GSH precursor glutathione reduced ethyl ester (GSHEE) effectively restores intracellular GSH levels in NK cells from acute leukemia models, thereby enhancing NK cell cytotoxicity and improving NK-cell-mediated antitumor immune responses [43].

##### Serine and other amino acids

Dietary serine restriction, which lowers systemic serine concentrations, impairs cytokine production in NK cells and suppresses NK-cell-mediated antitumor immune responses [45]. Accordingly, serine supplementation represents a potential strategy to restore NK cell antitumor function. However, given the dual role of serine in regulating NK cells, *Eubacterium rectale* transplantation, which consumes serine in the environment, could also be a novel approach to enhancing NK cell antitumor capacity [46]. Moreover, exogenous leucine supplementation sustains CD16-triggered IFN-γ expression in NK cells, thereby enhancing NK cell functionality [97].

##### Polyamines

Exogenous polyamine supplementation can enhance NK-cell-mediated antitumor immune responses. Addition of exogenous polyamines has been shown to restore metabolic flux, IFN-γ and GZMB production, and overall functionality in SREBP-deficient NK cells [51]. Supplementation with spermidine also increases expression of GZMB and IFN-γ in NK cells [50].

#### Lipids

In general, limiting lipid accumulation can enhance NK-cell-mediated antitumor immune responses. High-fat diets have been demonstrated to compromise NK cell immune function through multiple mechanisms. Therefore, restricting dietary fat intake may help sustain NK cell functionality [54]. Moreover, depletion of lipids within ascitic fluid restores expression of GZMB, IFN-γ, and TNF-α in NK cells, thereby augmenting NK cell cytotoxicity and overall antitumor efficacy [52,74]. Reducing lipid uptake by NK cells can also enhance their antitumor immune responses. Inhibition of the phospholipid uptake receptor scavenger receptor class B member 1 (SR-B1) has been demonstrated to up-regulate the expression of GZMB and perforin in most NK cells, thereby promoting cytotoxicity in vitro [74]. Furthermore, IL-1β blockade can suppress neutral lipid accumulation and secretion by pulmonary macrophages, diminish subsequent lipid deposition within lung NK cells, and restore their effector molecules expression along with cytotoxicity in the murine breast cancer models [75].

Supplementation with specific type of lipids can also enhance NK-cell-mediated antitumor immune responses. Exogenous administration of cholesterol notably enhances NK cell CD107a expression and cytotoxicity over the range of 10 to 100 μg·ml^−1^, while no further enhancement is observed at concentrations approaching physiological serum levels (approximately 1,000 to 2,000 μg·ml^−1^), indicating a concentration-dependent immunomodulatory role of cholesterol with optimal effects at moderate levels [68].

Moreover, decreasing PGE_2_ abundancy in the TME by inhibiting the metabolism of arachidonic acid to PGE_2_ can also enhance NK-cell-mediated antitumor immune responses. Inhibition of COX-2 with celecoxib in cocultures of acute myeloid leukemia (AML) blasts and NK cells prevents AML-induced suppression of NK cell effector function [62]. Similarly, administration of another COX-2 inhibitor, NS398, also effectively restores NK cell functionality impaired by exogenous PGE_2_ [58]. Furthermore, novel material-based strategies have also been developed to improve the pharmacological performance of COX-2 inhibitors, such as COX-2-inhibitor-based nanomedicine formulations [98].

### Targeting functional molecules involved in extracellular-metabolite-mediated NK cell regulation

**Fig. 6.**
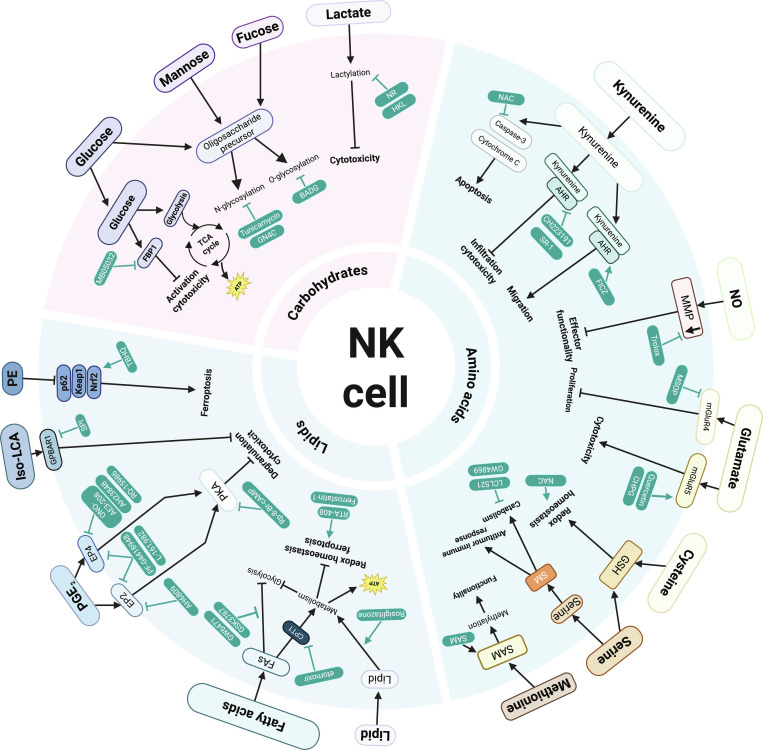
Metabolite-based modulation therapy by targeting associated functional molecules. Extracellular metabolites trigger a series of alterations within natural killer (NK) cells, including transcription, posttranslation modification, metabolism, redox homeostasis, and signal cascades. By either inhibiting or activating these functional molecules and corresponding alterations, the antitumor functionality of NK cells can be enhanced.

Extracellular metabolites regulate NK cells through multiple mechanisms, including activating receptors and triggering downstream signaling pathways, entering NK cells and serving as metabolic substrates or modification donors, and modulating the expression and activity of function-related proteins or pathways within NK cells. Targeting these metabolite-induced molecular alterations offers a potential strategy to enhance NK-cell-mediated antitumor immune responses (Fig. 6).

#### Carbohydrates and their metabolites

Enhancing the ability of NK cells to utilize glucose can improve their antitumor immune responses. Inhibition of fructose-1,6-bisphosphatase (FBP1) by MB05032 restores NK cell glycolytic flux, thereby augmenting cytokine-induced NK cell activation and cytotoxicity, contributing to tumor suppression [99].

Targeting NK cell glycosylation also represents an effective approach to enhancing their antitumor functionality. Inhibition of O-glycosylation by benzyl-2-acetoamido-2-deoxy-α-d-galactopyranoside (BADG) or blockade of N-glycosylation by *N*-acetyl-d-glucosamine-calix[4]arene (GN4C) has been shown to enhance NK cell cytotoxicity [10]. Likewise, tunicamycin, an inhibitor of N-glycosylation, suppresses N-glycosylation of CD16 and increases its affinity for immunoglobulin G, thereby potentiating ADCC of NK cells [100]. Tunicamycin also prevents the lysosomal localization of cystatin F through inhibition of N-glycosylation, thereby increasing cathepsin C activity, promoting perforin and granzyme activation [13].

In addition, targeting NK cell lactylation can improve their antitumor immune responses. Supplementation with nicotinamide riboside (NR) can reduce lactylation level in NK cells, thus up-regulating the expression of activating receptors such as NKG2D, CD38, and CD160 and potentiating NK cell cytotoxicity and tumor-killing capacity. Furthermore, combination treatment of NR with honokiol (HKL), which directly activates NAD-dependent protein deacetylase sirtuin-3 (SIRT3)-mediated delactylation, leads to a more pronounced decrease in NK cell lactylation level and a more substantial suppression of tumor burden, along with distinct prolongation of survival in tumor-bearing mice [22].

#### Amino acids and their metabolites

##### Kynurenine

Targeting ROS can enhance NK-cell-mediated antitumor immune responses. Treatment of NK cells with the antioxidant *N*-acetylcysteine (NAC) prevents cytochrome c release and caspase-3 activation, thereby protecting NK cells from kynurenine-induced apoptosis [26].

Targeting the AHR represents another promising approach to counteract kynurenine-mediated NK cell suppression. AHR antagonist CH223191 facilitates NK cell differentiation, increases the proportion of mature NK cells expressing CD94, TRAIL, and CD56 and up-regulates the expression of activating receptors such as NKG2D and NKp46, consequently restoring NK cell infiltration and cytotoxic activity [31]. Inhibition of AHR using StemRegenin-1 (SR-1) also alleviates oxidative stress and restores NK cell effector functions [28]. However, both AHR inhibition and activation can influence NK cell function, as the AHR agonist 6-formylindolo[3,2-b]carbazole (FICZ) has also been shown to enhance NK cell migration [30]. Therefore, the selection between AHR antagonists and agonists for therapeutic modulation of NK cell antitumor function warrants further investigation.

##### Arginine-NO axis

NO induces MMP loss and apoptosis in NK cells, impairing their effector functionality. Treatment with vitamin E analog Trolox partially restores the NO-induced decline in MMP of NK cells [36], implicating the role for antioxidants in maintaining mitochondrial homeostasis and alleviating NO-mediated NK cell dysfunction.

##### Glutamine-glutamate axis

Selective modulation of mGluRs represents an effective strategy to enhance NK cell functionality. Specifically, activation of mGluR5 by quercetin or chlorohydroxyphenylglycine (CHPG) enhances the cytotoxicity of NK cells [40], whereas inhibition of mGluR4 with (RS)-a-methylserine-*O*-phosphate (MSOP) increases the number of intratumoral NK cells and suppresses tumor growth in male mice [41]. Intravenous delivery of MSOP via nanoparticles further improves its bioavailability and tumor-targeting efficiency, augmenting its antitumor efficacy [41].

##### Cysteine-GSH Axis

GSH deficiency disrupts redox homeostasis within NK cells, leading to impaired NK cell functionality. Treatment with the antioxidant NAC effectively restores redox balance in NK cells from patients with acute leukemia, thereby enhancing NK-cell-mediated cytotoxicity and suppressing tumor growth [43].

##### Serine and other amino acids

Serine regulates NK cell functionality by serving as a precursor for SM synthesis. Selective inhibition of SM catabolism in NK cell membranes using the acidic sphingomyelinase inhibitor LCL521 and the neutral sphingomyelinase inhibitor GW4869 increases the number of protrusions on intratumoral NK cells, elevates their expression of IFN-γ, CD107a, and perforin, and markedly enhances NK-cell-mediated antitumor immune response both in vivo and in vitro [44].

Methionine participates in the regulation of NK cell methylation dynamics, and exogenous SAM supplementation can effectively restore IFN-γ secretion and cytotoxicity of NK cells [49].

#### Lipids

Enhancing lipid utilization capacity of NK cells can improve their antitumor immune responses. Pharmacological activation of PPARγ by the agonist rosiglitazone enhances lipid utilization in NK cells and markedly increases their IFN-γ production, although it fails to delay disease progression or reduce lymphoma burden [53].

Targeting FA-induced metabolic reprogramming also represents an effective approach to improving NK-cell-mediated antitumor immune response. Pharmacological inhibition of PPARα/δ prevents FA-driven metabolic reprogramming, thereby reversing NK cell metabolic paralysis and restoring their cytotoxicity following FFA exposure [54]. Using etomoxir to inhibit carnitine palmitoyltransferase 1 (CPT1), the enzyme responsible for FA transport, also restores the metabolic reprogramming toward glycolysis in NK cells, thereby reinstating their ability to polarize the MTOC and lytic granules to the synapse, ultimately recovering NK cell cytotoxicity [54].

Restoring redox homeostasis disrupted by lipids can also enhance NK-cell-mediated antitumor immune responses. Pharmacological activation of the NRF2 antioxidant pathway by RTA-408 or inhibition of ferroptosis with ferrostatin-1 effectively alleviates lipid-peroxidation-driven oxidative stress, thereby improving redox homeostasis and antitumor functionality of NK cells [52].

Targeting PGE_2-_activated EP receptors provides another promising avenue to enhance NK cell antitumor immune response. Pharmacological inhibition of EP4 using ONO-AE3-208 and AH23848 restores IFN-γ production and cytotoxicity of NK cells suppressed by PGE_2_ [59]. In animal models, EP4 antagonists AH23848 and RQ-15986 also reduce both spontaneous breast tumor metastasis and pulmonary colonization in an NK-cell-dependent manner [101]. Likewise, the EP2 antagonist AH6809 reverses PGE_2_-induced suppression of NK cell cytotoxicity [59]. Blockade of EP2/4 signaling with PF-04418948 and L-161982 further prevents the down-regulation of activating receptor NKG2D and restores cytokine production, as well as degranulation capacity of NK cells, when cocultured with AML blasts [62]. Since EP receptors also activates PKA, which suppresses proximal activation events in cytotoxic lymphocytes, inhibition of PKA I using Rp-8-Br-cAMP also counteracts the PGE_2_-mediated suppression of NK cell degranulation and cytotoxicity [61].

In addition, targeting iso-LCA and PE-related pathways can further enhance NK cell functionality. Application of GPBAR1 antagonist spironolactone (SPI) counteracted iso-LCA-induced suppression of p-CREM signaling, thereby reducing tumor burden in mice [73]. Likewise, treatment of Nrf2-specific agonist *tert*-butylhydroquinone (TBHQ) effectively reverses the suppression induced by PE, protecting NK cells from ferroptosis and increasing IL-2, IFN-γ, and TNF-α production [77].

### Combination with adoptive NK cell transfer therapy

Metabolite-based modulation therapy comprises strategies that modulate extracellular metabolite availability, as well as those targeting functional molecules involved in metabolite-mediated NK cell regulation. Beyond monotherapy, these approaches can also be integrated with other therapeutic modalities to enhance efficacy.

**Fig. 7.**
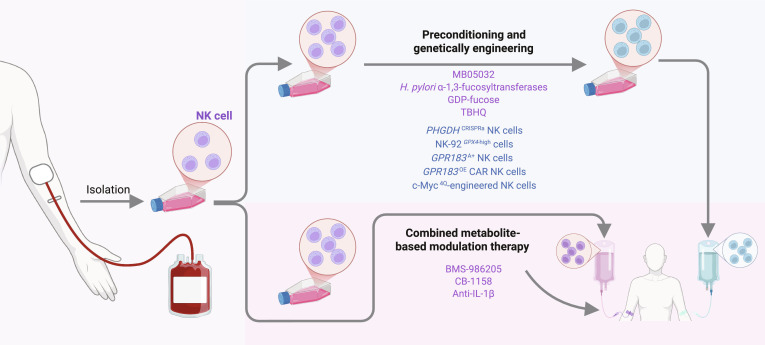
Metabolite-based modulation therapy in combination with adoptive natural killer (NK) cell transfer therapy. Combining metabolite-based modulation therapy with adoptive NK cell transfer therapy can achieve synergic effects. Before transfer, NK cells can be pretreated with therapeutic agents or genetically engineered, enabling them to better adapt to the harsh conditions of tumor microenvironment (TME). Alternatively, therapeutic agents can be administered concurrently with adoptive NK cell transfer therapy to remodel the TME. Thereby, the antitumor functionality of transferred NK cells is restored.

Adoptive NK cell transfer therapy represents an advanced avenue of cancer immunotherapy that enhances the immune system's capacity to recognize and eradicate malignant cells by isolating, modifying, and expanding NK cells derived from patients or donors before reinfusion. Given the distinct antitumor properties of NK cells, this therapeutic approach has emerged as a promising strategy for treating diverse malignancies. However, several studies have reported that adoptive NK cell transfer therapy shows limited efficacy against solid tumors, as NK cells often exhibit restricted infiltration into tumors and become functionally impaired within TME, thereby losing their antitumor activity [75]. In this context, metabolite-based modulation therapy can help restore NK cell functionality within TME and reinvigorate the antitumor potential of adoptively transferred NK cells (Fig. 7).

#### Preconditioning and genetically engineering NK cells before adoptive transfer

Preconditioning and genetic engineering of NK cells constitute effective strategies to augment their cytotoxicity before adoptive transfer.

Inhibition of FBP1 with MB05032 improves glucose utilization in NK cells, and adoptive transfer of MB05032-preconditioned NK cells markedly slows tumor growth in vivo [99]. Incubating T-cell-depleted BM progenitors with *Helicobacter pylori* α-1,3-fucosyltransferases and guanosine diphosphate fucose can generate ex vivo fucosylated NK cells, and recipients of these cells exhibit profound antitumor responses along with prolonged survival, accompanied by increased NK cell accumulation within lymphoma-involved organs, compared with those receiving unmodified NK cells [102]. Moreover, compared to untreated NK cells, adoptive transfer of NK cells activated by the Nrf2-specific agonist TBHQ considerably suppresses CRC liver metastasis, underscoring the value of counteracting PE-mediated inhibition to enhance the antitumor function of NK cells [77].

Genetically modifying NK cells prior to adoptive transfer represents another promising strategy to enhance their resilience against metabolic stress within the TME. Under serine-restricted conditions, conventional adoptive NK cell transfer fails to alleviate tumor burden or prolong survival in murine melanoma models. In contrast, transfer of NK cells whose *phosphoglycerate dehydrogenase (PHGDH)* expression is induced by electroporation of messenger RNA encoding a dCas9-VPR-based CRISPRa and single guide RNAs targeting the *PHGDH* promoter (*PHGDH* ^CRISPRa^), whose serine biosynthesis is up-regulated, distinctly reduces tumor size and extends survival in mice fed a serine-restricted diet [45]. Similarly, *GPX4*-overexpressing NK-92 cells (NK-92^*GPX4*-high^) exhibit resistance to kynurenine-induced cell death and display enhanced intratumoral infiltration and tumor suppression than control groups [27]. Moreover, intravenous transfer of either *GPR183*^A+^ NK-92 cells or *GPR183*^OE^ chimeric antigen receptor (CAR) NK-92 cells into NOD-*scid* IL2Rgamma^null^ (NSG) mice bearing MDA-MB-231 xenografts notably delays tumor growth compared to controls [72]. Beyond single-target modifications, engineering approaches targeting broader metabolic regulators have also demonstrated efficacy. c-Myc^4Q^-engineered NK cells, characterized by enhanced c-Myc transcription and protein stability, exhibit markedly improved metabolic adaptability and elevated effector molecule expression, markedly suppress tumor growth, and prolong survival in tumor-bearing mice [64].

#### Incorporating metabolite-based modulation therapy during adoptive NK cell transfer

The therapeutic efficacy of adoptive NK cell transfer can also be augmented through the concurrent application of metabolite-based modulation therapy.

Pharmacologic inhibition of IDO1 with BMS-986205 attenuates the immunosuppressive impact of IDO1 on the cytotoxicity of adoptively transferred GD2.CAR-NK cells, enhancing their antitumor functionality [103]. Likewise, in CT26 tumor-bearing mice, coadministration of the ARG inhibitor CB-1158 with NK cells distinctly reduced pulmonary metastases compared with adoptive NK cell transfer monotherapy [90]. IL-1β induces neutral lipid accumulation and secretion of pulmonary macrophages, which, in turn, promotes intracellular lipid deposition in NK cells and impairs their cytotoxic function. While either IL-1β blockade or adoptive NK cell transfer alone confers moderate therapeutic benefits, their combined administration yields a synergistic and potent antitumor response, reducing metastatic burden by approximately 7-fold [75].

### Combination with other therapies

**Fig. 8.**
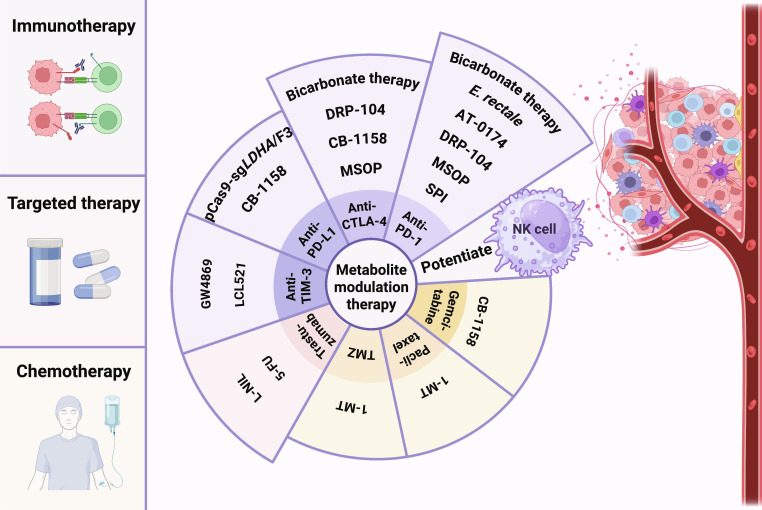
Metabolite-based-modulation therapy in combination with other therapeutic modalities. By integrating metabolite-based modulation therapy with immunotherapy, targeted therapy, and chemotherapy, the antitumor efficacy is further reinforced, suggesting promising potential for clinical combination strategies.

Metabolite-based modulation therapy can also integrate with other strategies, including chemotherapy, immunotherapy, and targeted therapy. Such combination regimens often achieve superior therapeutic efficacy compared with single-agent treatments, through either cooperative or complementary mechanisms (Fig. 8).

#### Combination with PD-1/PD-L1 blockade

Compared with monotherapy, the incorporation of bicarbonate therapy with anti- programmed cell death protein 1 (PD-1) antibody markedly improves therapeutic outcomes in pancreatic carcinoma and bicarbonate-resistant B16 melanoma models [104]. Codelivery of IDO1/TDO2 inhibitor AT-0174 along with anti-PD-1 antibody also elicits a more potent antitumor response in cisplatin-resistant non-small-cell lung cancer models compared with monotherapy [105]. Likewise, the glutamine antagonist DRP-104, when used in combination with anti-PD-1 antibody, improves survival and durable remission in colon cancer models [95]. Similarly, the mGluR4 antagonist MSOP paired with anti-PD-1 therapy more effectively restrains the growth of B16, MC38, and 3LL tumors [41]. In addition, *E. rectale* administration substantially enhances the therapeutic efficacy of anti-PD-1 treatment and prolongs the survival of anti-PD-1-treated mice [46]. Furthermore, iso-LCA antagonist SPI, when partnered with PD-1 blockade, markedly impedes hepatocellular carcinoma progression [73].

When it comes to anti-PD-L1 therapy, co-treatment of lipoplex pCas9-sg*LDHA*/F3 and anti-PD-L1 antibody elicits synergistic antitumor effect and prolonged survival of tumor-bearing mice [83]. Moreover, combination use of ARG inhibitor CB-1158 and anti-PD-L1 antibody also achieves a pronounced suppression of tumor expansion [90], further demonstrating the synergistic effect between metabolite-based modulation therapy and anti-PD-L1 antibody treatment.

#### Combination with other immunotherapeutic strategies

Metabolite-based modulation therapy offers broad opportunities to reinforce the efficacy of diverse immunotherapies beyond anti-PD-1/PD-L1 therapy, including anti-cytotoxic T-lymphocyte-associated protein 4 (CTLA-4) therapy, anti-T-cell immunoglobulin and mucin-domain containing-3 (TIM-3) therapy, and other immunomodulators.

In preclinical models, bicarbonate therapy enhances anti-CTLA-4 efficacy in bicarbonate-resistant B16 melanoma [104]. Likewise, the mGluR4 antagonist MSOP coapplied with anti-CTLA-4 antibodies further strengthens the suppression of B16, MC38, and 3LL tumor growth [41]. Furthermore, concurrent administration of ARG inhibitor CB-1158 with dual anti-PD-1 and anti-CTLA-4 antibodies markedly reduces primary tumor burden and diminishes pulmonary metastases across diverse cancer models [90]. Administration of glutamine antagonist DRP-104 alongside anti-CTLA-4 or anti-T cell immunoreceptor with immunoglobulin and immunoreceptor tyrosine-based inhibitory motif domains (TIGIT) antibody also improves survival of tumor-bearing mice and induces durable tumor regression [95]. When combined with anti-TIM-3 antibodies, both neutral sphingomyelinase inhibitor GW4869 and acidic sphingomyelinase inhibitor LCL521 notably enhance NK-cell-mediated cytotoxicity and achieve superior tumor suppression effect compared with monotherapy in vivo [44].

#### Combination with targeted therapy

Metabolite-based modulation therapy complements targeted therapies by alleviating metabolic suppression and remodeling the TME, thereby enhancing therapeutic responsiveness. 5-FU augments the efficacy of trastuzumab by depleting MDSCs and attenuating their ARG/iNOS-mediated inhibition of NK cell cytotoxicity, which consequently reduced tumor burden in murine models [35]. Similarly, when administrated together with iNOS inhibitor L-NIL, trastuzumab results in a marked reduction in tumor volume [35].

#### Combination with chemotherapy

Metabolite-based modulation therapy can potentiate the antitumor effects of conventional chemotherapeutics agents. For instance, oral administration of IDO inhibitor 1-MT along with paclitaxel markedly enhances the therapeutic efficacy of paclitaxel [106], and concurrent treatment of 1-MT with temozolomide (TMZ) also exhibits synergistic antitumor activity [107]. Similarly, combining ARG inhibitor CB-1158 with gemcitabine markedly suppresses tumor growth in CT26, LLC, and 4T1 models [90].

### Translational prospects and challenges

Beyond the preclinical evidence discussed above, numerous clinical trials have evaluated metabolite-based modulation therapies. While the majority of these studies did not specifically assess NK-cell-related outcomes, several studies have investigated the association between metabolic interventions and NK cell functional improvement.

Arginine supplementation represents the most extensively studied intervention in this context. Enhanced NK cell cytotoxicity following arginine supplementation has been observed in patients with breast cancer receiving chemotherapy and patients with CRC after surgical resection [108,109]. Similarly, glutamine-enriched nutritional therapy during chemotherapy in patients with acute lymphoblastic leukemia has been shown to increase the proportion of circulating NK cells [110]. In addition, as adoptive NK cell transfer emerges as a promising immunotherapeutic approach, clinical trials integrating this strategy with metabolite-based modulation therapies are also being conducted, including combination therapy with 5-FU (NCT05040438) and glycoengineering of adoptively transferred NK cells (ChiCTR2000041024).

Given the regulatory roles of extracellular metabolites on NK cell antitumor capacity, metabolite-based modulation therapy represents a promising therapeutic strategy. However, several key considerations merit particular attention during clinical translation.

First, ensuring drug safety is paramount. Given the ubiquity of metabolic processes throughout the body, interventions lacking tumor specificity may inadvertently disrupt normal physiological metabolism. ARG inhibitors, for example, enhance NK cell antitumor capacity by reducing extracellular arginine degradation. However, ARG also participates in the hepatic urea cycle, which is essential for ammonia detoxification, and systemic ARG inhibition may consequently precipitate hepatotoxicity [90]. Furthermore, nonspecific exposure to nanocarrier-delivered drugs may also lead to chronic inflammation, tissue injury, or immunotoxicity [111]. Therefore, strategies enabling TME-specific targeting would offer a superior safety profile. Alternatively, rigorous safety monitoring is warranted in the clinical implementation of metabolite-based modulation therapy to mitigate the risk of severe adverse events.

Second, translation efforts should be informed by evidence of therapeutic efficacy in clinical settings. Unlike in vitro experiments, the human body functions as an interconnected system, and different malignancies possess distinct characteristics. IDO1 inhibitors have been shown to reduce kynurenine production and thereby relieve its suppressive effects on NK cells. However, in patients with advanced urothelial carcinoma, addition of the selective IDO1 inhibitor epacadostat to the PD-1 inhibitor pembrolizumab failed to improve progression-free survival or overall survival compared to pembrolizumab alone [112], suggesting that other effects of IDO1 inhibition may counteract its enhancement of NK cell antitumor capacity or that urothelial carcinoma may be inherently insensitive to IDO1 inhibition. Therefore, given that preclinical and clinical trial outcomes do not always align, rigorous clinical validation is imperative prior to clinical implementation. Such validation not only confirms therapeutic efficacy but also informs subsequent drug optimization based on clinical findings.

Finally, pharmacokinetic considerations and standardization of clinical administration protocols warrant careful attention. While dietary or nutritional supplementation constitutes a common and relatively safe approach to metabolic modulation, exogenously administered nutrients are subject to rapid metabolism, and variations in dosage and administration frequency can substantially influence therapeutic outcomes [108,113]. Accordingly, both drug design and clinical trial protocols should be oriented toward practical clinical applicability, with particular emphasis on ensuring effective and sustained delivery of therapeutic agents.

Taken together, as a therapeutic approach that has demonstrated preliminary potential in cancer treatment, metabolite-based modulation therapy holds considerable translational promise and warrants further clinical investigation, while careful attention must be given to drug safety, efficacy, and standardization of administration protocols during translational process.

## Conclusion and Perspectives

With the expanding understanding of tumor-immune interactions, the multifaceted functions and regulatory mechanisms of NK cells in tumors have attracted substantial interest. As a crucial component of TME, extracellular metabolites play pivotal roles in directly remodeling NK cell antitumor biology. They not only act as metabolic substrates or energetic fuels but also function as signaling mediators that modulate NK cell activation, transcriptional programs, and effector responses, thereby collectively modulating NK-cell-mediated antitumor immunity. Despite accumulating evidence has underscored the importance of these metabolic cues in regulating NK cell function, the regulatory mechanisms within have yet to be systematically elucidated.

In this review, we highlight the direct regulatory effects of carbohydrates, amino acids, lipids, and their corresponding metabolites on NK cell antitumor immune response. These metabolites regulate NK cell functionality through multiple mechanisms, including functioning as structural components, providing metabolic substrates, inducing metabolic reprogramming, serving as substrates for protein and RNA modifications, regulating gene transcription, maintaining stress homeostasis, engaging receptors and triggering downstream signaling cascades, modulating intracellular pH, among others. Furthermore, as an emerging area of interest, gut-microbiota-derived metabolites have also been implicated in regulating NK cell antitumor functions. Microbiota-derived butyrate has been reported to enhance NK cell viability, cytotoxicity, and migration [114], while another study demonstrated that butyrate restricts NK cell function by down-regulating their metabolic capacity [115]. Such contradictory findings are not unique to butyrate, and many other metabolites are found to exert opposing effects on NK cell function through distinct mechanisms. For example, FAs can serve as metabolic fuel to enhance NK cell activity [116] yet can also bind to PPARs and induce metabolic reprogramming of NK cells, which diminishes their glycolytic capacity and impairs antitumor function [56]. This phenomenon underscores the complexity of metabolite-mediated regulation of NK cells, as NK cells at distinct developmental stages, activation states, or tissue locations may respond differently to the same stimulus through distinct mechanisms or with varying sensitivities. Furthermore, in addition to direct regulation of NK cells, extracellular metabolites can also modulate NK cell antitumor function indirectly by acting on other cell types. For instance, fructose enhances CAF proliferation and activation [117], while CAFs promote NK cell migration into stromal areas and down-regulate their activating receptor expression, ultimately impairing NK cell antitumor capacity [118]. Therefore, advancing methodological innovations in NK cell functional analysis and refined investigations into NK cell biology are essential to precisely delineate how metabolites shape the antitumor immune response of NK cell.

Given that functionality and antitumor response of NK cells are always impaired by extracellular metabolites within the immunosuppressive TME, restoration of NK cell activity can be achieved by modulating the availability of metabolites in the TME or by targeting associated signaling pathways. Specifically, the antitumor response of NK cells can be enhanced through several strategies including (a) altering dietary intake or adopting alternative strategies to alter the concentrations of metabolites within the TME; (b) targeting metabolic enzymes to adjust metabolite catabolism and metabolic flux, thereby modulating the level of downstream metabolites; (c) modulating enzymes responsible for metabolite secretion or transport to reshape their spatial distribution within the TME; and (d) targeting metabolite-induced signaling pathways within NK cells to restore their activation and cytotoxic capacity. Furthermore, these metabolite-based modulation approaches can be effectively integrated with other therapeutic modalities, not only exerting complementary effects but also generating synergistic benefits.

Notably, several critical issues regarding the overall efficacy and clinical translation of metabolite-based modulation therapy warrant careful consideration. TME functions as an integrated system, encompassing not only NK cells but also tumor cells and various immune or stromal populations such as T cells, dendritic cells, and CAFs. Therefore, alterations in the levels of metabolites within the TME can also influence these cells, which may, in turn, affect the overall therapeutic effect. An illustrative example of synergistic antitumor activity following treatment is that COX-2 inhibitors can enhance NK-cell-mediated antitumor immune response by reducing environmental PGE2 production, concurrently suppress tumor cell proliferation, augment CD8^+^ T-cell-mediated cytotoxicity, inhibit macrophage’s polarization toward the M2 phenotype, and promote the recruitment and activation of dendritic cells [98]. Consequently, these pleiotropic but intrinsically synergic effects contribute to the potent antitumor efficacy of COX-2 inhibitors. An example of antagonistic antitumor effects following treatment can be observed with glutamine metabolism inhibitor. Although functionality of NK cells is largely independent of glutamine metabolism, both tumor cells and T cells within the TME are highly dependent on it to sustain their proliferation and activities [119,120], and the application of glutaminase inhibitors will not only suppress tumor growth but also compromise T-cell-mediated antitumor immune responses, yielding complex effects. Therefore, the overall therapeutic outcome of metabolite-based modulation therapy represents a subtle balance among multiple interacting factors within the TME.

Moreover, while dysregulation of specific metabolite dominates the impact on NK cell antitumor function in certain contexts, the metabolite profile within the TME is typically a complicated and interconnected network. For instance, it remains challenging to discern which specific component in the tumor interstitial fluid medium is responsible for inducing proteostasis imbalance and functional impairment in human NK cells [121]. Furthermore, given the integrative nature of human physiology, systemic supplementation or modulation of metabolites could inevitably affect multiple organs, and responses of these nontumor organs may, in turn, influence the overall therapeutic effects within tumor-infiltrating organs. Hence, a holistic perspective is essential when using metabolite-based modulation therapy to enhance NK cell antitumor function, and the ultimate clinical efficacy warrants validation through large-scale cohort trials.

In summary, NK cell functionality within the TME is intricately shaped by diverse metabolites through multiple regulatory mechanisms. Therapeutic strategies targeting these metabolic cues in the TME represent a promising avenue for restoring NK cell antitumor immune response and, when combined with other modalities, can yield superior antitumor efficacy. Hence, the complicated interplay between NK cells and tumor warrants continued attention and investigation. With the ongoing advancement of NK-cell-centered research, it is anticipated that the therapeutic potential of NK cells will benefit a broader range of patients with cancer and provide valuable insights into lifestyle and dietary interventions for cancer prevention and management.

## References

[1] Chen S, Zhu H, Jounaidi Y. Comprehensive snapshots of natural killer cells functions, signaling, molecular mechanisms and clinical utilization. Signal Transduct Target Ther. 2024;9(1):302. 39511139 10.1038/s41392-024-02005-w PMC11544004

[2] Vivier E, Rebuffet L, Narni-Mancinelli E, Cornen S, Igarashi RY, Fantin VR. Natural killer cell therapies. Nature. 2024;626(8000):727–736. 38383621 10.1038/s41586-023-06945-1

[3] Ran GH, Lin YQ, Tian L, Zhang T, Yan DM, Yu JH, Deng YC. Natural killer cell homing and trafficking in tissues and tumors: From biology to application. Signal Transduct Target Ther. 2022;7(1):205. 35768424 10.1038/s41392-022-01058-z PMC9243142

[4] Das A, Ding Y, Harly C, Bhandoola A. The development of innate lymphoid cells. Nat Immunol. 2026;27:401–412. 41634488 10.1038/s41590-025-02414-1 PMC12959067

[5] Lin A, Ye P, Li Z, Jiang A, Liu Z, Cheng Q, Zhang J, Luo P. Natural killer cell immune checkpoints and their therapeutic targeting in cancer treatment. Research. 2025;8:0723. 40463500 10.34133/research.0723 PMC12131497

[6] Keating SE, Zaiatz-Bittencourt V, Loftus RM, Keane C, Brennan K, Finlay DK, Gardiner CM. Metabolic reprogramming supports IFN-γ production by CD56^bright^ NK cells. J Immunol. 2016;196(6):2552–2560. 26873994 10.4049/jimmunol.1501783

[7] Assmann N, O’Brien KL, Donnelly RP, Dyck L, Zaiatz-Bittencourt V, Loftus RM, Heinrich P, Oefner PJ, Lynch L, Gardiner CM, et al. Srebp-controlled glucose metabolism is essential for NK cell functional responses. Nat Immunol. 2017;18(11):1197–1206. 28920951 10.1038/ni.3838

[8] Ehlers FAI, Mahaweni NM, Olieslagers TI, Bos GMJ, Wieten L. Activated natural killer cells withstand the relatively low glucose concentrations found in the bone marrow of multiple myeloma patients. Front Oncol. 2021;11: Article 622896. 34094908 10.3389/fonc.2021.622896 PMC8174784

[9] Glasner A, Roth Z, Varvak A, Miletic A, Isaacson B, Bar-On Y, Jonjic S, Khalaila I, Mandelboim O. Identification of putative novel O-glycosylations in the NK killer receptor Ncr1 essential for its activity. Cell Discov. 2015;1:15036. 27462433 10.1038/celldisc.2015.36 PMC4860851

[10] Yan C, Lu P, Jiang Y, Miao S, Zhao L, Xu X. 2B4/CD244 signaling in immune regulation and its role in infection, cancer, and immune tolerance. ImmunoTargets Ther. 2025;14:1111–1131. 41070200 10.2147/ITT.S538126 PMC12506779

[11] Shao Y, Zhang Y, Cao J, He J, He Q, Lei Y, Tang N, Zhou Y. NKp30: A key membrane molecule in the fight against cancer and infection. FASEB J. 2025;39(14): Article e70856. 40678939 10.1096/fj.202501490R PMC12272517

[12] Patel KR, Nott JD, Barb AW. Primary human natural killer cells retain proinflammatory IgG1 at the cell surface and express CD16a glycoforms with donor-dependent variability. Mol Cell Proteomics. 2019;18(11):2178–2190. 31467031 10.1074/mcp.RA119.001607 PMC6823852

[13] Senjor E, Pirro M, Švajger U, Prunk M, Sabotič J, Jewett A, Hensbergen PJ, Perišić Nanut M, Kos J. Different glycosylation profiles of cystatin F alter the cytotoxic potential of natural killer cells. Cell Mol Life Sci. 2023;81(1):8. 38092995 10.1007/s00018-023-05041-x PMC10719177

[14] Sudholz H, Meng X, Park HY, Shen Z, Nikolic I, Cursons J, Goddard-Borger ED, Schuster IS, Andoniou CE, Degli-Esposti MA, et al. Core fucosylation of IL-2RB is required for natural killer cell homeostasis. Cell Rep. 2025;44(8): Article 116101. 40753573 10.1016/j.celrep.2025.116101

[15] Feinberg D, Ramakrishnan P, Wong DP, Asthana A, Parameswaran R. Inhibition of O-GlcNAcylation decreases the cytotoxic function of natural killer cells. Front Immunol. 2022;13: Article 841299. 35479087 10.3389/fimmu.2022.841299 PMC9036377

[16] Dong Z, Yuan Z, Jin T, Gao C, Wang X, Xu F. Lactate at the crossroads of tumor metabolism and immune escape: A new frontier in cancer therapy. J Transl Med. 2025;23(1):1239. 41204256 10.1186/s12967-025-07272-x PMC12595878

[17] Brand A, Singer K, Koehl GE, Kolitzus M, Schoenhammer G, Thiel A, Matos C, Bruss C, Klobuch S, Peter K, et al. LDHA-associated lactic acid production blunts tumor immunosurveillance by T and NK cells. Cell Metab. 2016;24:657–671. 27641098 10.1016/j.cmet.2016.08.011

[18] Brand A, Singer K, Koehl GE, Kolitzus M, Schoenhammer G, Thiel A, Matos C, Bruss C, Klobuch S, Peter K, et al. Decreased LDHB expression in breast tumor cells causes NK cell activation and promotes tumor progression. Cancer Biol Med. 2024;21:513–540. 38525901 10.20892/j.issn.2095-3941.2023.0382 PMC11208901

[19] Harmon C, Robinson MW, Hand F, Almuaili D, Mentor K, Houlihan DD, Hoti E, Lynch L, Geoghegan J, O’Farrelly C. Lactate-mediated acidification of tumor microenvironment induces apoptosis of liver-resident NK cells in colorectal liver metastasis. Cancer Immunol Res. 2019;7(2):335–346. 30563827 10.1158/2326-6066.CIR-18-0481

[20] Varner EL, Trefely S, Bartee D, von Krusenstiern E, Izzo L, Bekeova C, O’Connor RS, Seifert EL, Wellen KE, Meier JL, et al. Quantification of lactoyl-CoA (lactyl-CoA) by liquid chromatography mass spectrometry in mammalian cells and tissues. Open Biol. 2020;10(9): Article 200187. 32961073 10.1098/rsob.200187 PMC7536085

[21] Tan Y, Tan W, Liang Y, Long Y, Chen S, Hu Q, Ou Y, Fu J, Chen H, Ren F, et al. Machine learning-enabled spatial multi-omics uncovers lactate-driven targets and tumor microenvironmental reprogramming in cancer. npj Digit Med. 2025;9:109. 41469480 10.1038/s41746-025-02286-7 PMC12864923

[22] Jin J, Yan P, Wang D, Bai L, Liang H, Zhu X, Zhu H, Ding C, Wei H, Wang Y. Targeting lactylation reinforces NK cell cytotoxicity within the tumor microenvironment. Nat Immunol. 2025;26(7):1099–1112. 40494934 10.1038/s41590-025-02178-8

[23] Juras PK, Racioppi L, Mukherjee D, Artham S, Gao X, Akullian D’Agostino L, Chang CY, McDonnell DP. Increased CaMKK2 expression is an adaptive response that maintains the fitness of tumor-infiltrating natural killer cells. Cancer Immunol Res. 2023;11(1):109–122. 36301267 10.1158/2326-6066.CIR-22-0391 PMC9812906

[24] Kern Coquillat N, Picq L, Hamond A, Megy P, Benezech S, Drouillard A, Lager-Lachaud N, Cahoreau E, Moreau M, Fallone L, et al. Pivotal role of exogenous pyruvate in human natural killer cell metabolism. Nat Metab. 2025;7(2):336–347. 39753710 10.1038/s42255-024-01188-4

[25] Du R, Zhang X, Lu X, Ma X, Guo X, Shi C, Ren X, Ma X, He Y, Gao Y, et al. PDPN positive CAFs contribute to HER2 positive breast cancer resistance to trastuzumab by inhibiting antibody-dependent NK cell-mediated cytotoxicity. Drug Resist Updat. 2023;68: Article 100947. 36812747 10.1016/j.drup.2023.100947

[26] Song H, Park H, Kim YS, Kim KD, Lee HK, Cho DH, Yang JW, Hur DY. L-kynurenine-induced apoptosis in human NK cells is mediated by reactive oxygen species. Int Immunopharmacol. 2011;11(8):932–938. 21352963 10.1016/j.intimp.2011.02.005

[27] Cui JX, Xu XH, He T, Liu JJ, Xie TY, Tian W, Liu JY. L-kynurenine induces NK cell loss in gastric cancer microenvironment via promoting ferroptosis. J Exp Clin Cancer Res. 2023;42(1):52. 36855135 10.1186/s13046-023-02629-w PMC9976385

[28] Trikha P, Moseman JE, Thakkar A, Campbell AR, Elmas E, Foltz JA, Chakravarti N, Fitch JR, Mardis ER, Lee DA. Defining the AHR-regulated transcriptome in NK cells reveals gene expression programs relevant to development and function. Blood Adv. 2021;5(22):4605–4618. 34559190 10.1182/bloodadvances.2021004533 PMC8759121

[29] Scoville SD, Nalin AP, Chen L, Chen L, Zhang MH, McConnell K, Beceiro Casas S, Ernst G, Traboulsi AA, Hashi N, et al. Human AML activates the aryl hydrocarbon receptor pathway to impair NK cell development and function. Blood. 2018;132(17):1792–1804. 30158248 10.1182/blood-2018-03-838474 PMC6202909

[30] Shin JH, Moreno-Nieves UY, Zhang LH, Chen C, Dixon AL, Linde MH, Mace EM, Sunwoo JB. AHR regulates NK cell migration via ASB2-mediated ubiquitination of filamin A. Front Immunol. 2021;12: Article 624284. 33717133 10.3389/fimmu.2021.624284 PMC7943850

[31] Park A, Yang Y, Lee Y, Kim MS, Park YJ, Jung H, Kim TD, Lee HG, Choi I, Yoon SR. Indoleamine-2,3-dioxygenase in thyroid cancer cells suppresses natural killer cell function by inhibiting NKG2D and NKp46 expression via STAT signaling pathways. Journal of. Clin Med. 2019;8(6):842. 10.3390/jcm8060842 PMC6617210 31212870

[32] Goh CC, Roggerson KM, Lee H-C, Golden-Mason L, Rosen HR, Hahn YS. Hepatitis C virus-induced myeloid-derived suppressor cells suppress NK cell IFN-γ production by altering cellular metabolism via arginase-1. J Immunol. 2016;196(5):2283–2292. 26826241 10.4049/jimmunol.1501881 PMC4761460

[33] Zhao R, He B, Huang L, Wu Y, Liu T, Liu J, Zhao M, Zhong T, Zhang Y, Zhang X, et al. Targeting AQP5-mediated arginine deprivation in gastric cancer stem cells restores NK cell anti-tumor immunity. Cell Rep Med. 2025;6(9): Article 102333. 40961922 10.1016/j.xcrm.2025.102333 PMC12490235

[34] Gazdic M, Simovic Markovic B, Jovicic N, Misirkic-Marjanovic M, Djonov V, Jakovljevic V, Arsenijevic N, Lukic ML, Volarevic V. Mesenchymal stem cells promote metastasis of lung cancer cells by downregulating systemic antitumor immune response. Stem Cells Int. 2017;2017(1):6294717. 28798777 10.1155/2017/6294717 PMC5534320

[35] Stiff A, Trikha P, Mundy-Bosse B, McMichael E, Mace TA, Benner B, Kendra K, Campbell A, Gautam S, Abood D, et al. Nitric oxide production by myeloid-derived suppressor cells plays a role in impairing fc receptor-mediated natural killer cell function. Clin Cancer Res. 2018;24(8):1891–1904. 29363526 10.1158/1078-0432.CCR-17-0691 PMC7184799

[36] Takabayashi A, Kawai Y, Iwata S, Kanai M, Denno R, Kawada K, Obama K, Taki Y. Nitric oxide induces a decrease in the mitochondrial membrane potential of peripheral blood lymphocytes, especially in natural killer cells. Antioxid Redox Signal. 2000;2(4):673–680. 11213472 10.1089/ars.2000.2.4-673

[37] Fang H, Dai W, Gu R, Zhang Y, Li J, Luo W, Tong S, Han L, Wang Y, Jiang C, et al. myCAF-derived exosomal PWAR6 accelerates CRC liver metastasis via altering glutamine availability and NK cell function in the tumor microenvironment. J Hematol Oncol. 2024;17(1):126. 39696364 10.1186/s13045-024-01643-5 PMC11657131

[38] Loftus RM, Assmann N, Kedia-Mehta N, O’Brien KL, Garcia A, Gillespie C, Hukelmann JL, Oefner PJ, Lamond AI, Gardiner CM, et al. Amino acid-dependent cMyc expression is essential for NK cell metabolic and functional responses in mice. Nat Commun. 2018;9(1):2341. 29904050 10.1038/s41467-018-04719-2 PMC6002377

[39] Kim H-H, Shim Y-R, Kim HN, Yang K, Ryu T, Kim K, Choi SE, Kim MJ, Woo C, Chung KP, et al. xCT-mediated glutamate excretion in white adipocytes stimulates interferon-γ production by natural killer cells in obesity. Cell Rep. 2023;42(6): Article 112636. 37310859 10.1016/j.celrep.2023.112636

[40] Choi W-M, Ryu T, Lee J-H, Shim Y-R, Kim M-H, Kim H-H, Kim YE, Yang K, Kim K, Choi SE, et al. Metabotropic glutamate receptor 5 in natural killer cells attenuates liver fibrosis by exerting cytotoxicity to activated stellate cells. Hepatology. 2021;74(4):2170–2185. 33932306 10.1002/hep.31875

[41] Wan Z, Sun R, Liu Y-W, Li S, Sun J, Li J, Zhu J, Moharil P, Zhang B, Ren P, et al. Targeting metabotropic glutamate receptor 4 for cancer immunotherapy. Sci Adv. 2021;7(50):eabj4226. 34890233 10.1126/sciadv.abj4226 PMC8664261

[42] Xia C, Xing X, Zhang W, Wang Y, Jin X, Tian M, Ba X, Hao F. Cysteine and homocysteine can be exploited by GPX4 in ferroptosis inhibition independent of GSH synthesis. Redox Biol. 2024;69: Article 102999. 38150992 10.1016/j.redox.2023.102999 PMC10829872

[43] Zhao Y, Wang Y, Liang T, Song X, Zhu Y, Liu X, Lv M, Zheng C, Ni F. Dysregulated glutathione metabolism impairs natural killer cell function in patients with acute leukemia. Int Immunopharmacol. 2025;154: Article 114566. 40184815 10.1016/j.intimp.2025.114566

[44] Zheng X, Hou Z, Qian Y, Zhang Y, Cui Q, Wang X, Shen Y, Liu Z, Zhou Y, Fu B, et al. Tumors evade immune cytotoxicity by altering the surface topology of NK cells. Nat Immunol. 2023;24(5):802–813. 36959292 10.1038/s41590-023-01462-9

[45] Li JH, Feng Q, Ball AB, Lee CD, Wallerius ML, Bormin JG, Kapelczak ED, Armstrong WR, Hermans L, Krall A, et al. Species-specific serine metabolism differentially controls natural killer cell functions. Nat Metab. 2025;7(9):1905–1923. 40813920 10.1038/s42255-025-01348-0 PMC12861115

[46] Liu N, Chen L, Yan M, Tao Q, Wu J, Chen J, Chen X, Zhang W, Peng C. *Eubacterium rectale* improves the efficacy of anti-PD1 immunotherapy in melanoma via l-serine-mediated NK cell activation. Research. 2023;6:0127. 37223471 10.34133/research.0127 PMC10202379

[47] Tassinari V, Jia W, Chen W-L, Candi E, Melino G. The methionine cycle and its cancer implications. Oncogene. 2024;43(48):3483–3488. 39394448 10.1038/s41388-024-03122-0

[48] Song H, Song J, Cheng M, Zheng M, Wang T, Tian S, Flavell RA, Zhu S, Li HB, Ding C, et al. METTL3-mediated m6A RNA methylation promotes the anti-tumour immunity of natural killer cells. Nat Commun. 2021;12(1):5522. 34535671 10.1038/s41467-021-25803-0 PMC8448775

[49] Meng M, Zhong Z, Song L, Zhang Z, Yin X, Xie X, Tian L, Wu W, Yang Y, Deng Y, et al. mTOR signaling promotes rapid m6A mRNA methylation to regulate NK-cell activation and effector functions. Cancer Immunol Res. 2024;12(8):1039–1057. 38640466 10.1158/2326-6066.CIR-23-0339

[50] He H, Song Z, Lin S, Wang Y, Wang G. Exploring the effect of polyamines on NK cell function in colorectal cancer process based on glycolysis. Int Immunopharmacol. 2023;117: Article 109944. 36871536 10.1016/j.intimp.2023.109944

[51] O’Brien KL, Assmann N, O’Connor E, Keane C, Walls J, Choi C, Oefner PJ, Gardiner CM, Dettmer K, Finlay DK. De novo polyamine synthesis supports metabolic and functional responses in activated murine NK cells. Eur J Immunol. 2021;51(1):91–102. 32946110 10.1002/eji.202048784

[52] Poznanski SM, Singh K, Ritchie TM, Aguiar JA, Fan IY, Portillo AL, Rojas EA, Vahedi F, El-Sayes A, Xing S, et al. Metabolic flexibility determines human NK cell functional fate in the tumor microenvironment. Cell Metab. 2021;33(6):1205–1220.e5. 33852875 10.1016/j.cmet.2021.03.023

[53] Kobayashi T, Lam PY, Jiang H, Bednarska K, Gloury R, Murigneux V, Tay J, Jacquelot N, Li R, Tuong ZK, et al. Increased lipid metabolism impairs NK cell function and mediates adaptation to the lymphoma environment. Blood. 2020;136(26):3004–3017. 32818230 10.1182/blood.2020005602

[54] Michelet X, Dyck L, Hogan A, Loftus RM, Duquette D, Wei K, Beyaz S, Tavakkoli A, Foley C, Donnelly R, et al. Metabolic reprogramming of natural killer cells in obesity limits antitumor responses. Nat Immunol. 2018;19(12):1330–1340. 30420624 10.1038/s41590-018-0251-7

[55] Gao X, Sun Z, Liu X, Luo J, Liang X, Wang H, Zhou J, Yang C, Wang T, Li J. 127aa encoded by circSpdyA promotes FA synthesis and NK cell repression in breast cancers. Cell Death Differ. 2025;32(3):416–433. 39402211 10.1038/s41418-024-01396-1 PMC11894148

[56] Hu X, Jia X, Xu C, Wei Y, Wang Z, Liu G, You Q, Lu G, Gong W. Downregulation of NK cell activities in apolipoprotein C-III-induced hyperlipidemia resulting from lipid-induced metabolic reprogramming and crosstalk with lipid-laden dendritic cells. Metabolism. 2021;120: Article 154800. 34051224 10.1016/j.metabol.2021.154800

[57] Pedde A-M, Kim H, Donakonda S, Baumann T, Bayerl F, Meiser P, Hirschberger A, Klement C, Grassmann S, Öllinger R, et al. Tissue-colonizing disseminated tumor cells secrete prostaglandin E2 to promote NK cell dysfunction and evade anti-metastatic immunity. Cell Rep. 2024;43(11): Article 114855. 39541209 10.1016/j.celrep.2024.114855

[58] Li T, Zhang Q, Jiang Y, Yu J, Hu Y, Mou T, Chen G, Li G. Gastric cancer cells inhibit natural killer cell proliferation and induce apoptosis via prostaglandin E2. Oncoimmunology. 2016;5(2): Article e1069936. 27057432 10.1080/2162402X.2015.1069936 PMC4801461

[59] Park A, Lee Y, Kim MS, Kang YJ, Park Y-J, Jung H, Kim TD, Lee HG, Choi I, Yoon SR. Prostaglandin E2 secreted by thyroid cancer cells contributes to immune escape through the suppression of natural killer (NK) cell cytotoxicity and NK cell differentiation. Front Immunol. 2018;9:1859. 30140269 10.3389/fimmu.2018.01859 PMC6094168

[60] Patterson C, Hazime KS, Zelenay S, Davis DM. Prostaglandin E_2_ impacts multiple stages of the natural killer cell antitumor immune response. Eur J Immunol. 2024;54(2): Article e2350635. 38059519 10.1002/eji.202350635

[61] Martinet L, Jean C, Dietrich G, Fournié J-J, Poupot R. PGE2 inhibits natural killer and gamma delta T cell cytotoxicity triggered by NKR and TCR through a cAMP-mediated PKA type I-dependent signaling. Biochem Pharmacol. 2010;80(6):838–845. 20470757 10.1016/j.bcp.2010.05.002

[62] Rothfuß C, Baumann T, Donakonda S, Brauchle B, Marcinek A, Urban C, Mergner J, Pedde AM, Hirschberger A, Krupka C, et al. Two-layered immune escape in AML is overcome by fcγ receptor activation and inhibition of PGE2 signaling in NK cells. Blood. 2025;145(13):1395–1406. 39840945 10.1182/blood.2024025706

[63] Holt D, Ma X, Kundu N, Fulton A. Prostaglandin E2 (PGE2) suppresses natural killer cell function primarily through the PGE2 receptor EP4. Cancer Immunol Immunother. 2011;60(11):1577–1586. 21681369 10.1007/s00262-011-1064-9 PMC3686482

[64] Jiao D, Sun R, Ren X, Wang Y, Tian P, Wang Y, Yuan D, Yue X, Wu Z, Li C, et al. Lipid accumulation-mediated histone hypoacetylation drives persistent NK cell dysfunction in anti-tumor immunity. Cell Rep. 2023;42(10): Article 113211. 37792534 10.1016/j.celrep.2023.113211

[65] Li JH, Zhou A, Lee CD, Shah SN, Ji JH, Senthilkumar V, Padilla ET, Ball AB, Feng Q, Bustillos CG, et al. MEF2C regulates NK cell effector functions through control of lipid metabolism. Nat Immunol. 2024;25(5):778–789. 38589619 10.1038/s41590-024-01811-2 PMC12135675

[66] Cheng Z, Gu J, Lu Y, Cai M, Zhang T, Wang J. Regulatory mechanisms of lipid rafts in remodeling the tumor immune microenvironment of colorectal cancer and targeted therapeutic strategies. Biomolecules. 2025;15(12):1675. 41463331 10.3390/biom15121675 PMC12730349

[67] Qin W-H, Yang Z-S, Li M, Chen Y, Zhao X-F, Qin Y-Y, Song J-Q, Wang B-B, Yuan B, Cui X-L, et al. High serum levels of cholesterol increase antitumor functions of nature killer cells and reduce growth of liver tumors in mice. Gastroenterology. 2020;158(6):1713–1727. 31972238 10.1053/j.gastro.2020.01.028

[68] Zhang Y, Wang Z, Lu Y, Sanchez DJ, Li J, Wang L, Meng X, Chen J, Kien TT, Zhong M, et al. Region-specific CD16^+^ neutrophils promote colorectal cancer progression by inhibiting natural killer cells. Adv Sci. 2024;11(29): Article e2403414. 10.1002/advs.202403414 PMC11304263 38790136

[69] Guo X-J, Zhu B-B, Li J, Guo P, Niu Y-B, Shi J-L, Yokoyama W, Huang Q-S, Shao D-Y. Cholesterol metabolism in tumor immunity: Mechanisms and therapeutic opportunities for cancer. Biochem Pharmacol. 2025;234: Article 116802. 39954742 10.1016/j.bcp.2025.116802

[70] Li Y, Orange JS. Degranulation enhances presynaptic membrane packing, which protects NK cells from perforin-mediated autolysis. PLoS Biol. 2021;19(8): Article e3001328. 34343168 10.1371/journal.pbio.3001328 PMC8330931

[71] Li W, Johnson H, Yuan X-M, Jonasson L. 7β-hydroxycholesterol induces natural killer cell death via oxidative lysosomal destabilization. Free Radic Res. 2009;43(11):1072–1079. 19707922 10.1080/10715760903176919

[72] Kim Y-M, Tsai MK, Sun C, Laveroni O, Akana RV, Frombach K, Jerby L. Engineering NK and T cells with metabolite-sensing receptors to target solid tumors. Nat Immunol. 2026;27(5):1039–1052. 41872506 10.1038/s41590-026-02473-y PMC13132725

[73] Wei H, Suo C, Gu X, Shen S, Lin K, Zhu C, Yan K, Bian Z, Chen L, Zhang T, et al. AKR1D1 suppresses liver cancer progression by promoting bile acid metabolism-mediated NK cell cytotoxicity. Cell Metab. 2025;37(5):1103–1118.e7. 40010348 10.1016/j.cmet.2025.01.011

[74] Slattery K, Yao C-H, Mylod E, Scanlan J, Scott B, Crowley JP, McGowan O, McManus G, Brennan M, O’brien K, et al. Uptake of lipids from ascites drives NK cell metabolic dysfunction in ovarian cancer. Sci Immunol. 2025;10(107):eadr4795. 40344087 10.1126/sciimmunol.adr4795

[75] Gong Z, Li Q, Shi J, Liu ET, Shultz LD, Ren G. Lipid-laden lung mesenchymal cells foster breast cancer metastasis via metabolic reprogramming of tumor cells and natural killer cells. Cell Metab. 2022;34(12):1960–1976.e9. 36476935 10.1016/j.cmet.2022.11.003 PMC9819197

[76] Liu X, Lu J, Ni X, He Y, Wang J, Deng Z, Zhang G, Shi T, Chen W. FASN promotes lipid metabolism and progression in colorectal cancer via the SP1/PLA2G4B axis. Cell Death Discov. 2025;11(1):122. 40148316 10.1038/s41420-025-02409-9 PMC11950308

[77] Cai R, Meng Y, Ru M, Wang X, Li W, Liu X, Zhuang S, Huang Y, Diao H. Ketogenic diet impairs NK cell cytotoxic function in colorectal cancer liver metastasis by inducing ferroptosis via suppression of the p62-Keap1-Nrf2 pathway. Redox Biol. 2026;89: Article 103969. 41385828 10.1016/j.redox.2025.103969 PMC12757549

[78] Lagadari M, Truta-Feles K, Lehmann K, Berod L, Ziemer M, Idzko M, Barz D, Kamradt T, Maghazachi AA, Norgauer J. Lysophosphatidic acid inhibits the cytotoxic activity of NK cells: Involvement of Gs protein-mediated signaling. Int Immunol. 2009;21:667–677. 19461126 10.1093/intimm/dxp035

[79] Bossowski JP, Pillai R, Kilian J, Wong Lau A, Nakamura M, Rashidfarrokhi A, Hao Y, Li R, Wu K, Hattori T, et al. The integrated stress response promotes immune evasion through lipocalin 2. Nature. 2026;652:1329–1338. 41708864 10.1038/s41586-026-10143-0 PMC13128482

[80] Chang C-H, Qiu J, O’Sullivan D, Buck MD, Noguchi T, Curtis JD, Chen Q, Gindin M, Gubin MM, Van Der Windt GJ, et al. Metabolic competition in the tumor microenvironment is a driver of cancer progression. Cell. 2015;162(6):1229–1241. 26321679 10.1016/j.cell.2015.08.016 PMC4864363

[81] Zhang W, Kim H-S, Park H-B, An E-K, Kim S-J, Ryu D, Kwak M, Zhang X, Xu J, Lee PC, et al. Fucoidan from *Durvillaea antarctica* elicits anticancer immunity against lung cancer via natural killer cell activation. Int J Biol Macromol. 2025;322: Article 146783. 40803462 10.1016/j.ijbiomac.2025.146783

[82] Ge W, Meng L, Cao S, Hou C, Zhu X, Huang D, Li Q, Peng Y, Jiang K. The SIX1/LDHA axis promotes lactate accumulation and leads to NK cell dysfunction in pancreatic cancer. J Immunol Res. 2023;2023(1):6891636. 36937004 10.1155/2023/6891636 PMC10022590

[83] Ju H, Kim D, Oh Y-K. Lipid nanoparticle-mediated CRISPR/Cas9 gene editing and metabolic engineering for anticancer immunotherapy. Asian J Pharm Sci. 2022;17(5):641–652. 36382304 10.1016/j.ajps.2022.07.005 PMC9640370

[84] Noble RA, Bell N, Blair H, Sikka A, Thomas H, Phillips N, Nakjang S, Miwa S, Crossland R, Rand V, et al. Inhibition of monocarboxyate transporter 1 by AZD3965 as a novel therapeutic approach for diffuse large B-cell lymphoma and Burkitt lymphoma. Haematologica. 2017;102(7):1247–1257. 28385782 10.3324/haematol.2016.163030 PMC5566036

[85] Chen J, Bu C, Lu Y, Peng X, Yu J, Ding X, Yuan P, Hong S. Bioresponsive nanoreactor initiates cascade reactions for tumor vascular normalization and lactate depletion to augment immunotherapy. Biomaterials. 2025;317: Article 123100. 39799700 10.1016/j.biomaterials.2025.123100

[86] Peng T, Shao X, Song W, Xu W, Xiong W, He Y, Ding Y, Huang Y. Intratumoral lactic acid neutralization strategy for boosting chemoimmunotherapy using liposomal sodium bicarbonate. Sci Bull. 2024;69(24):3936–3948. 10.1016/j.scib.2024.08.042 39547906

[87] Wang Z, Li W, Jiang Y, Tran TB, Cordova LE, Chung J, Kim M, Wondrak G, Erdrich J, Lu J. Sphingomyelin-derived nanovesicles for the delivery of the IDO1 inhibitor epacadostat enhance metastatic and post-surgical melanoma immunotherapy. Nat Commun. 2023;14(1):7235. 37945606 10.1038/s41467-023-43079-4 PMC10636136

[88] Angka L, Tanese de Souza C, Baxter KE, Khan ST, Market M, Martel AB, Tai LH, Kennedy MA, Bell JC, Auer RC. Perioperative arginine prevents metastases by accelerating natural killer cell recovery after surgery. Mol Ther. 2022;30(10):3270–3283. 35619558 10.1016/j.ymthe.2022.05.024 PMC9552810

[89] Ohashi T, Akazawa T, Aoki M, Kuze B, Mizuta K, Ito Y, Inoue N. Dichloroacetate improves immune dysfunction caused by tumor-secreted lactic acid and increases antitumor immunoreactivity. Int J Cancer. 2013;133(5):1107–1118. 23420584 10.1002/ijc.28114

[90] Steggerda SM, Bennett MK, Chen J, Emberley E, Huang T, Janes JR, Li W, MacKinnon AL, Makkouk A, Marguier G, et al. Inhibition of arginase by CB-1158 blocks myeloid cell-mediated immune suppression in the tumor microenvironment. J Immunother Cancer. 2017;5(1):101. 29254508 10.1186/s40425-017-0308-4 PMC5735564

[91] Doshi AS, Cantin S, Hernandez M, Srinivasan S, Tentarelli S, Griffin M, Wang Y, Pop-Damkov P, Prickett LB, Kankkonen C, et al. Novel arginase inhibitor, AZD0011, demonstrates immune cell stimulation and antitumor efficacy with diverse combination partners. Mol Cancer Ther. 2023;22(5):630–645. 36912782 10.1158/1535-7163.MCT-22-0431

[92] Martí i Líndez AA, Reith W. Arginine-dependent immune responses. Cell Mol Life Sci. 2021;78(13):5303–5324. 34037806 10.1007/s00018-021-03828-4 PMC8257534

[93] Kaufmann Y, Kornbluth J, Feng Z, Fahr M, Schaefer RF, Klimberg VS. Effect of glutamine on the initiation and promotion phases of DMBA-induced mammary tumor development. JPEN J Parenter Enteral Nutr. 2003;27(6):411–418. 14621122 10.1177/0148607103027006411

[94] Edwards DN, Ngwa VM, Raybuck AL, Wang S, Hwang Y, Kim LC, Cho SH, Paik Y, Wang Q, Zhang S, et al. Selective glutamine metabolism inhibition in tumor cells improves antitumor T lymphocyte activity in triple-negative breast cancer. J Clin Invest. 2021;131(4): Article e140100. 33320840 10.1172/JCI140100 PMC7880417

[95] Yokoyama Y, Estok TM, Wild R. Sirpiglenastat (DRP-104) induces antitumor efficacy through direct, broad antagonism of glutamine metabolism and stimulation of the innate and adaptive immune systems. Mol Cancer Ther. 2022;21(10):1561–1572. 35930753 10.1158/1535-7163.MCT-22-0282

[96] Sinha R, Sinha I, Calcagnotto A, Trushin N, Haley JS, Schell TD, Richie JP. Oral supplementation with liposomal glutathione elevates body stores of glutathione and markers of immune function. Eur J Clin Nutr. 2018;72(1):105–111. 28853742 10.1038/ejcn.2017.132 PMC6389332

[97] De Federicis D, Capuano C, Ciuti D, Molfetta R, Galandrini R, Palmieri G. Nutrient transporter pattern in CD56^dim^ NK cells: CD16 (FcγRIIIA)-dependent modulation and association with memory NK cell functional profile. Front Immunol. 2024;15:1477776. 39606236 10.3389/fimmu.2024.1477776 PMC11599182

[98] Huang R, Yu J, Zhang B, Li X, Liu H, Wang Y. Emerging COX-2 inhibitors-based nanotherapeutics for cancer diagnosis and treatment. Biomaterials. 2025;315: Article 122954. 39549439 10.1016/j.biomaterials.2024.122954

[99] Cong J, Wang X, Zheng X, Wang D, Fu B, Sun R, Tian Z, Wei H. Dysfunction of natural killer cells by FBP1-induced inhibition of glycolysis during lung cancer progression. Cell Metab. 2018;28(2):243–255.e5. 30033198 10.1016/j.cmet.2018.06.021

[100] Drescher B, Witte T, Schmidt RE. Glycosylation of FcγRIII in N163 as mechanism of regulating receptor affinity. Immunology. 2003;110(3):335–340. 14632661 10.1046/j.1365-2567.2003.01743.x PMC1783064

[101] Ma X, Holt D, Kundu N, Reader J, Goloubeva O, Take Y, Fulton AM. A prostaglandin E (PGE) receptor EP4 antagonist protects natural killer cells from PGE2-mediated immunosuppression and inhibits breast cancer metastasis. Oncoimmunology. 2013;2(1): Article e22647. 23482441 10.4161/onci.22647 PMC3583931

[102] Tong X, Ru Y, Fu J, Wang Y, Zhu J, Ding Y, Lv F, Yang M, Wei X, Liu C, et al. Fucosylation promotes cytolytic function and accumulation of NK cells in B cell lymphoma. Front Immunol. 2022;13: Article 904693. 35784355 10.3389/fimmu.2022.904693 PMC9240281

[103] Caforio M, Sorino C, Caruana I, Weber G, Camera A, Cifaldi L, De Angelis B, Del Bufalo F, Vitale A, Goffredo BM, et al. GD2 redirected CAR T and activated NK-cell-mediated secretion of IFNγ overcomes MYCN-dependent IDO1 inhibition, contributing to neuroblastoma cell immune escape. J Immunother Cancer. 2021;9(3): Article e001502. 33737337 10.1136/jitc-2020-001502 PMC7978286

[104] Pilon-Thomas S, Kodumudi KN, El-Kenawi AE, Russell S, Weber AM, Luddy K, Damaghi M, Wojtkowiak JW, Mulé JJ, Ibrahim-Hashim A, et al. Neutralization of tumor acidity improves antitumor responses to immunotherapy. Cancer Res. 2016;76(6):1381–1390. 26719539 10.1158/0008-5472.CAN-15-1743 PMC4829106

[105] Wu C, Spector SA, Theodoropoulos G, Nguyen DJM, Kim EY, Garcia A, Savaraj N, Lim DC, Paul A, Feun LG, et al. Dual inhibition of IDO1/TDO2 enhances anti-tumor immunity in platinum-resistant non-small cell lung cancer. Cancer Metab. 2023;11(1):7. 37226257 10.1186/s40170-023-00307-1 PMC10207715

[106] Yoshida N, Ino K, Ishida Y, Kajiyama H, Yamamoto E, Shibata K, Terauchi M, Nawa A, Akimoto H, Takikawa O, et al. Overexpression of indoleamine 2,3-dioxygenase in human endometrial carcinoma cells induces rapid tumor growth in a mouse xenograft model. Clin Cancer Res. 2008;14(22):7251–7259. 19010841 10.1158/1078-0432.CCR-08-0991

[107] Hanihara M, Kawataki T, Oh-Oka K, Mitsuka K, Nakao A, Kinouchi H. Synergistic antitumor effect with indoleamine 2,3-dioxygenase inhibition and temozolomide in a murine glioma model. J Neurosurg. 2016;124(6):1594–1601. 26636389 10.3171/2015.5.JNS141901

[108] Angka L, Martel AB, Ng J, Pecarskie A, Sadiq M, Jeong A, Scaffidi M, Tanese de Souza C, Kennedy MA, Tadros S, et al. A translational randomized trial of perioperative arginine imunonutrition on natural killer cell function in colorectal cancer surgery patients. Ann Surg Oncol. 2022;29(12):7410–7420. 35879482 10.1245/s10434-022-12202-y

[109] Brittenden J, Heys SD, Ross J, Park KG, Eremin O. Natural cytotoxicity in breast cancer patients receiving neoadjuvant chemotherapy: Effects of L-arginine supplementation. Eur J Surg Oncol. 1994;20(4):467–472. 8076711

[110] Han Y, Zhang F, Wang J, Zhu Y, Dai J, Bu Y, Yang Q, Xiao Y, Sun X. Application of glutamine-enriched nutrition therapy in childhood acute lymphoblastic leukemia. Nutr J. 2016;15(1):65. 27401338 10.1186/s12937-016-0187-4 PMC4940940

[111] Sahu K, Satapathy T, Sahu P, Chandrakar O. Nanocarrier-induced inflammation: Mechanisms, immunometabolic impacts and strategies for safer design. TransMed. 2026;1(1): Article 100002.

[112] Long GV, Dummer R, Hamid O, Gajewski TF, Caglevic C, Dalle S, Arance A, Carlino MS, Grob JJ, Kim TM, et al. Epacadostat plus pembrolizumab versus placebo plus pembrolizumab in patients with unresectable or metastatic melanoma (ECHO-301/KEYNOTE-252): A phase 3, randomised, double-blind study. Lancet Oncol. 2019;20(8):1083–1097. 31221619 10.1016/S1470-2045(19)30274-8

[113] Zhang C, Chen J, Huai X, Wang X, Tao Y, Zhou S, Xuan W, Qian G, Tian J. An analysis of research on healthy lifestyle and digestive system cancers using bibliometric methods over the last two decades. TransMed. 2026;1(1): Article 100012.

[114] Mu S, Chang M, Shen Y, Wu X, Han Y, Xiang H, Luo Y, Chen Y, Zheng H, Song Z, et al. Gut microbiota metabolite butyric acid alleviated *Klebsiella Pneumoniae* induced lung injury by regulating CX3CR1^+^NK via PI3K/AKT pathway. Burns Trauma. 2026;14:tkaf069. 41532069 10.1093/burnst/tkaf069 PMC12794618

[115] Zaiatz-Bittencourt V, Jones F, Tosetto M, Scaife C, Cagney G, Jones E, Doherty GA, Ryan EJ. Butyrate limits human natural killer cell effector function. Sci Rep. 2023;13(1):2715. 36792800 10.1038/s41598-023-29731-5 PMC9932090

[116] Schimmer S, Mittermüller D, Werner T, Görs PE, Meckelmann SW, Finlay DK, Dittmer U, Littwitz-Salomon E. Fatty acids are crucial to fuel NK cells upon acute retrovirus infection. Front Immunol. 2023;14:1296355. 38094304 10.3389/fimmu.2023.1296355 PMC10716207

[117] Cui Y, Liu H, Zhang L, Zhang H, Wang Z, Wang J, Wang Z, Song L, Guo H, Liu L, et al. Fructose drives colorectal cancer progression by regulating crosstalk between cancer-associated fibroblasts and tumour cells. Gut. 2025;75(3):548–562. 10.1136/gutjnl-2025-335014 40935617

[118] Ben-Shmuel A, Gruper Y, Halperin C, Levi-Galibov O, Rosenberg-Fogler H, Barki D, Carradori G, Stein Y, Yagel G, Naumova M, et al. Cancer-associated fibroblasts serve as decoys to suppress NK cell anticancer cytotoxicity in breast cancer. Cancer Discov. 2025;15(6):1247–1269. 40052789 10.1158/2159-8290.CD-24-0131

[119] Shah R, Singh SJ, Eddy K, Filipp FV, Chen S. Concurrent targeting of glutaminolysis and metabotropic glutamate receptor 1 (GRM1) reduces glutamate bioavailability in GRM1^+^ melanoma. Cancer Res. 2019;79(8):1799–1809. 30987979 10.1158/0008-5472.CAN-18-1500 PMC6469683

[120] Buck MD, O’Sullivan D, Pearce EL. T cell metabolism drives immunity. J Exp Med. 2015;212(9):1345–1360. 26261266 10.1084/jem.20151159 PMC4548052

[121] Ji JH, Armstrong WR, Li JH, Lalani A, Lee CD, Bharadwaj V, Ho K, Liang J, Muir A, O’Sullivan TE. The transcriptional repressor Fli1 inhibits proteostasis during nutrient stress to limit NK cell persistence in solid tumors. Immunity. 2026;59(3):717–733. 41713422 10.1016/j.immuni.2026.01.017

